# Machine Learning-Based Predictions of Henry Coefficients for Long-Chain Alkanes in One-Dimensional Zeolites: Application to Hydroisomerization

**DOI:** 10.1021/acs.jpcc.5c03868

**Published:** 2025-09-26

**Authors:** Shrinjay Sharma, Ping Yang, Yachan Liu, Kevin Rossi, Peng Bai, Marcello S. Rigutto, Erik Zuidema, Umang Agarwal, Richard Baur, Sofia Calero, David Dubbeldam, Thijs J.H. Vlugt

**Affiliations:** † Process and Energy Department, Faculty of Mechanical Engineering, 2860 Delft University of Technology, Leeghwaterstraat 39, 2628CB Delft, The Netherlands; ‡ Department of Applied Physics and Science Education, Eindhoven University of Technology, 5600MB Eindhoven, The Netherlands; § Department of Chemical Engineering, 542579 University of Massachusetts Amherst, Amherst, Massachusetts 01003, United States; ∥ Department of Materials Science and Engineering, Faculty of Mechanical Engineering, Delft University of Technology, Mekelweg 2, 2628CD Delft, The Netherlands; ⊥ 100493 Shell Global Solutions International B.V., Grasweg 39, 1031HW Amsterdam, The Netherlands; # Shell Chemical LP, Monaca, Pennsylvania 15061, United States; ∇ Van’t Hoff Institute of Molecular Sciences, 1234 University of Amsterdam, Science Park 904, 1098XH Amsterdam, The Netherlands; ○ Climate Safety and Security Centre, TU Delft The Hague Campus, Delft University of Technology, 2594 AC The Hague, The Netherlands

## Abstract

Shape-selective adsorption in zeolites plays a pivotal role in catalytic hydroisomerization of long-chain alkanes, a key process in producing sustainable aviation fuels from Fischer–Tropsch products. Accurately predicting adsorption behavior for the large number of alkane isomers in different zeolite frameworks is computationally intensive. To address this, we have developed a machine learning framework that rapidly and accurately predicts Henry coefficients of linear (C_1_–C_30_) and branched (C_4_–C_20_) alkanes in one-dimensional zeolites. Using descriptors based on chain length, branching patterns, and molecular graphs, we evaluate multiple ML models, including Random Forest, XGBoost, CatBoost, TabPFN, and D-MPNN in MTT-, MTW-, MRE-, and AFI-type zeolites. TabPFN and D-MPNN offer the highest predictive accuracy. Active learning further boosts model performance by efficiently selecting diverse and structurally informative isomers. We also uncover activity cliffs, where small changes in molecular structure lead to sharp variations in adsorption, and demonstrate that targeted oversampling of these cases improves model robustness. Finally, we combine the ML-predicted Henry coefficients with gas-phase thermodynamics to compute reaction equilibrium distributions for C_16_ hydroisomerization. This integrated, data-driven approach enables efficient screening and design of shape-selective zeolite catalysts, thereby reducing the need for costly simulations.

## Graphical abstract

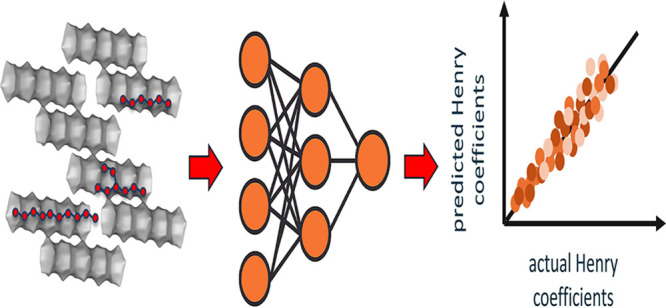

## 1 Introduction

In transitioning toward producing fuels and chemicals from renewable sources, platforms that deliver clean hydrocarbon liquid energy carriers, either directly from carbon dioxide or via biobased intermediates, are expected to play a significant role.^1^ For applications such as sustainable aviation fuel, low-carbon gas oils and lubricants, iso-alkanes with a high degree of branching are the preferred components due to favorable combustion and flow properties.^2^ Consequently, shape-selective zeolite-catalyzed hydroisomerization, commonly referred to as catalytic dewaxing, is going to be a crucial step in the production of branched alkanes, just as it is in the manufacturing of conventional petroleum-derived analogues.^3^

Predicting selectivities at reaction equilibrium for hydroisomerization, particularly at low conversion, requires accurate knowledge of both gas phase thermochemical properties and adsorption behavior inside zeolite pores. In our recent study on shape selectivity of zeolites on hydroisomerization,^4^ we demonstrated that combining the gas phase Gibbs free energies and the enthalpies of formation and Henry coefficients inside zeolite pores enables reliable prediction of isomer selectivities at reaction equilibrium. This can be very useful for determining the product distribution for long chain alkanes in zeolites, which are difficult to obtain from experiments. While thermochemical properties of long chain alkanes in the gas phase can be reliably predicted using our previously developed linear regression model based on second-order group contributions,^5^ obtaining accurate Henry coefficients still remains a major challenge. Henry coefficients are essential for understanding adsorption-based shape selectivities of branched and linear alkanes in zeolite frameworks, providing insight into how molecular structures influence zeolite adsorption. The Henry coefficient is defined as the slope of the adsorption isotherm at low loading in units of mol/kg framework/Pa. Henry coefficients are usually obtained from force field-based molecular simulations^6^ which are computationally demanding, particularly for a large number of isomers for long chain alkanes. This makes large-scale screening impractical, especially when enumerating hundreds of thousands of isomers.^7^ This calls for the need to develop robust and accurate Machine Learning (ML) models for quick and reliable predictions of Henry coefficients.

As the alkane chain length increases, the number of possible structural isomers grows exponentially^7^ due to the large number of ways carbon atoms can be arranged. For instance, C_4_ has only two isomers, C_10_ has 75, and C_20_ has over 366,000 isomers.^7^ This rapid combinatorial explosion presents a significant challenge for systematic studies involving long-chain alkanes. To efficiently explore this large chemical space, it is essential to implement automated enumeration of isomers and generation of the corresponding SMILES (Simplified Molecular Input Line Entry System) strings.^8^ SMILES representations are essential for simulations, property predictions, and machine learning workflows. Several tools are available for isomer enumeration, each with distinct strengths and limitations. PubChemPy^9,10^ supports chemical searches but not full isomer enumeration. MOLGEN (closed-source) and MAYGEN (open-source)^11^ reliably generate comprehensive sets of structural isomers. Surge is another open source isomer generator which uses a canonical path method for enumeration.^12^ ENU,^7^ a graph theory-based tool optimized for acyclic alkane isomers, is particularly efficient for long-chain hydrocarbons. As shown in Table 1, the SMILES strings generated by ENU^7^ do not always follow IUPAC naming conventions. This complicates the systematic classification of isomers, an important step in understanding zeolite shape selectivity for hydroisomerization. To address this issue, we have developed an isomer enumeration code in C++ and Python to generate exhaustive lists of alkane isomers containing methyl, ethyl, propyl, and isopropyl branches. Isomers with branches larger than propyl or isopropyl groups are excluded, as such bulky substituents are unlikely to adsorb in the narrow pores (ca. 5–6 Å)^13^ of one-dimensional zeolites.

**Table 1:** Comparison of SMILES Strings Obtained from ENU Software^7^ and the SMILES Strings^8^ Corresponding to IUPAC Nomenclature^14^ for Different Isomers

IUPAC abbreviation	SMILES (ENU software)	SMILES (IUPAC)
2,3,9-m-7-e-C_10_	CCC(CCCC(C)C(C)C)CC(C)C	CC(C)C(C)CCCC(CC)CC(C)C
2,3,8-m-6-p-C_10_	CCCC(CCC(C)C(C)C)CC(C)C	CC(C)C(C)CCC(CCC)CC(C)C

Henry coefficients for alkanes are typically computed using the Widom particle insertion method^15^ combined with the Configurational Bias Monte Carlo (CBMC) algorithm.^16,17^ This method becomes computationally expensive for large numbers of structural isomers of long-chain alkanes, for a large number of zeolite frameworks. ML models have recently emerged as a promising alternative for predicting Henry coefficients. These models have gained significant importance for accelerating materials discovery and optimization for a range of applications, such as gas separation, adsorption-based storage, and catalyst design.^18^ Recent reviews have highlighted the transformative potential of AI in zeolite discovery and adsorption modeling, including generative design, property prediction, and simulation acceleration.^19,20^ In porous materials such as Metal–Organic Frameworks (MOFs) and zeolites, ML has proven to be highly effective in predicting adsorption properties, thereby enabling high throughput screening without resorting to computationally expensive molecular simulations or time-consuming experiments.^21–24^ Efficient ML models have been developed to predict temperature-dependent Henry coefficients and adsorption selectivities for various adsorbates in MOFs, facilitating rapid evaluation for gas separation applications with small molecules such as CO_2_, CH_4_, and H_2_ capture.^25^ A comprehensive study by Gharagheizi and Sholl^26^ systematically evaluates the accuracy of IAST in predicting binary gas adsorption for a wide range of porous materials and conditions. Daou et al.^27^ combined ML with Ideal Adsorbed Solution Theory (IAST) to efficiently screen silica and cationic zeolites for short chain alkane separation. These authors used ML predicted isotherms to identify trends in capture storage and selectivity trends. Beyond adsorption selectivities, other studies have examined the underlying structure–property relationships. In particular, Rzepa et al.^18^ revealed that adsorption enthalpy and entropy correlate linearly in various zeolite–adsorbate systems, governed largely by the degree of molecular confinement. Liu et al.^28^ have developed a three-dimensional Convolutional Neural Network (CNN)^29^ framework ZeoNet designed to predict Henry coefficients for n-octadecane adsorption in over 330,000 zeolite structures using efficient grid-based volumetric representations. This method significantly outperforms traditional geometric-descriptor models and achieves near-simulation accuracy while being orders of magnitude faster.^28^ Despite these advances, systematic studies of the entire long-chain alkane space, especially in the context of zeolite-based adsorption, remain insufficiently investigated. The main challenge lies in the large number of zeolites (ca. 10^6^ hypothetical structures^30^) and the millions of possible isomers for long-chain alkanes.^7^ Computing Henry coefficients for such a large number of alkane–zeolite combinations using molecular simulations is practically not feasible. Our work addresses this gap by exploring ML-based prediction of Henry coefficients for long chain alkane isomers in one-dimensional zeolites, specifically MTT-, MTW-, MRE-, and AFI-type zeolites (Figure 1) because of the simplicity of the structures, its relevance for hydroisomerization reactions, and the absence of complicated window effects.^31,32^ While this study focuses on training and evaluating models within individual zeolite frameworks, future work will explore cross-framework generalization to assess model transferability for different pore geometries and confinement environments. Prediction of Henry coefficients of alkanes is particularly important for hydroisomerization applications, where adsorption-based shape selectivity governs catalytic performance.

**Figure 1:**
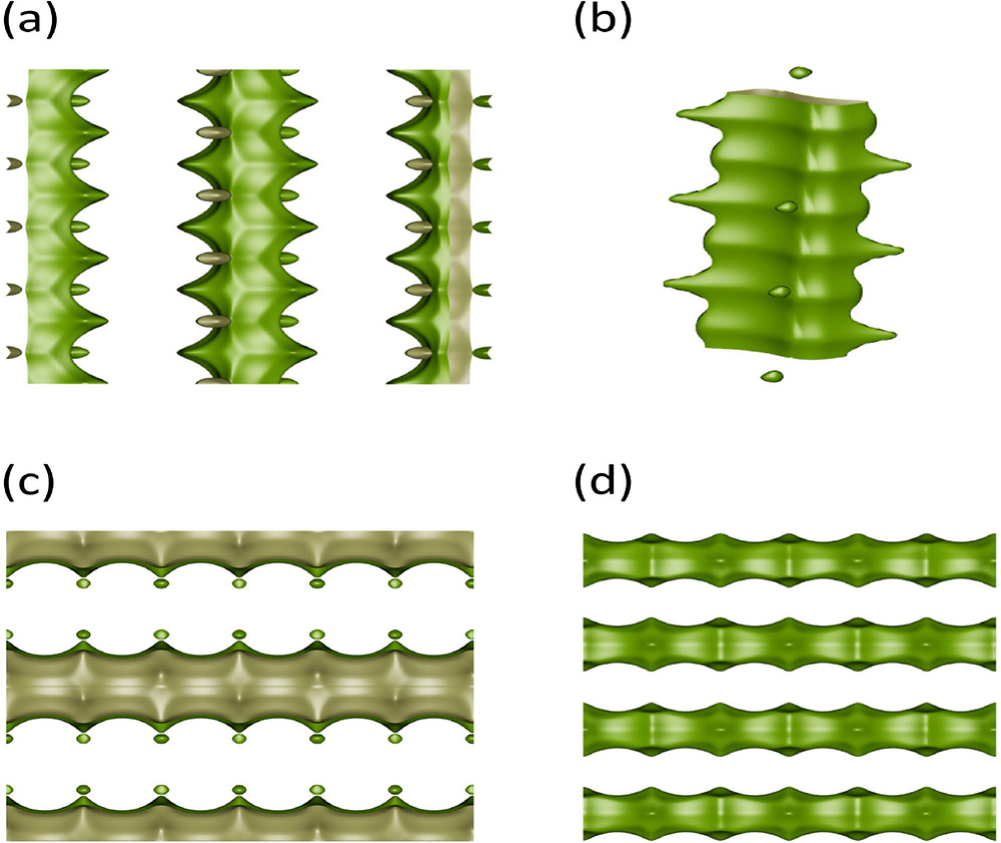
Typical representations of pore structures of one-dimensional zeolites: (a) MTW-type zeolite, (b) MTT-type zeolite, (c) MRE-type zeolite, and (d) AFI-type zeolite. The iRASPA software^33^ is used to generate these images.

A major challenge here arises from activity cliffs,^34,35^ which are sharp discontinuities in structure–property relationships, where minor structural changes yield disproportionately large differences in molecular properties. Activity cliffs occur because adsorption in narrow-pore zeolites is highly sensitive to alkyl branching. These cliffs are particularly problematic for ML models, which often assume a smooth structure–property landscape.^35^ Though extensively studied in medicinal chemistry, activity cliffs have not been addressed in the context of hydrocarbon adsorption to the best of our knowledge. In the case of alkane adsorption in zeolites, activity cliffs are observed in Henry coefficients due to effects such as branching position, symmetry, and confinement-induced steric interactions. A small structural change like a methyl group shift from one position to the next in branched alkanes can significantly alter its fit in one-dimensional zeolite pores, leading to sharp variations in Henry coefficients. For example, 2,3- and 2,4-dimethylpentane (2,3-m-C_5_ and 2,4-m-C_5_) isomers in MRE-type zeolite at 500 K differ by orders of magnitude in Henry coefficients (9.44 × 10^–8^ and 4.85 × 10^–6^ mol/kg framework/Pa, respectively).^4^ This is due to the shape of the zeolite pores. The corrugations in the zeolite pores arise from alternating peaks and crests caused by the arrangement of the zeolite atoms.^4^ This results in variations in channel diameter over the length of the pore (Figure 2). The likelihood of the methyl branches in 2,4-m-C_5_ fitting into two separate peaks is higher than for 2,3-m-C_5_ (Figure 2). This is due to the larger separation between the methyl groups in 2,4-m-C_5_, which allows for more favorable adsorption in the corrugated pore structure. The most widely used metric to identify such cliffs is the Structure–Activity Landscape Index (SALI),^36^ defined as the ratio of the absolute property difference and a structural distance metric. Typical examples of structural distance metrics are Tanimoto coefficient^37,38^ and Levenshtein distance^39^ between SMILES strings. Alternative approaches are two- and three-dimensional activity cliff maps,^36,40^ Matched Molecular Pair (MMP) analysis,^41^ and gradient-based landscape profiling.^42^ Several recent studies have emphasized the importance of explicitly identifying and incorporating activity cliff data into training pipelines through structural diversity sampling, active learning, and contrastive learning strategies.^43–45^ We also quantify activity cliffs for long-chain alkanes using Levenshtein distance-based SALI indices and systematically analyze the impact on ML model accuracy. This provides not only a performance benchmark for conventional and graph-based models, but also a strategy for improving predictive power in chemically diverse and cliff-prone data sets.

**Figure 2:**
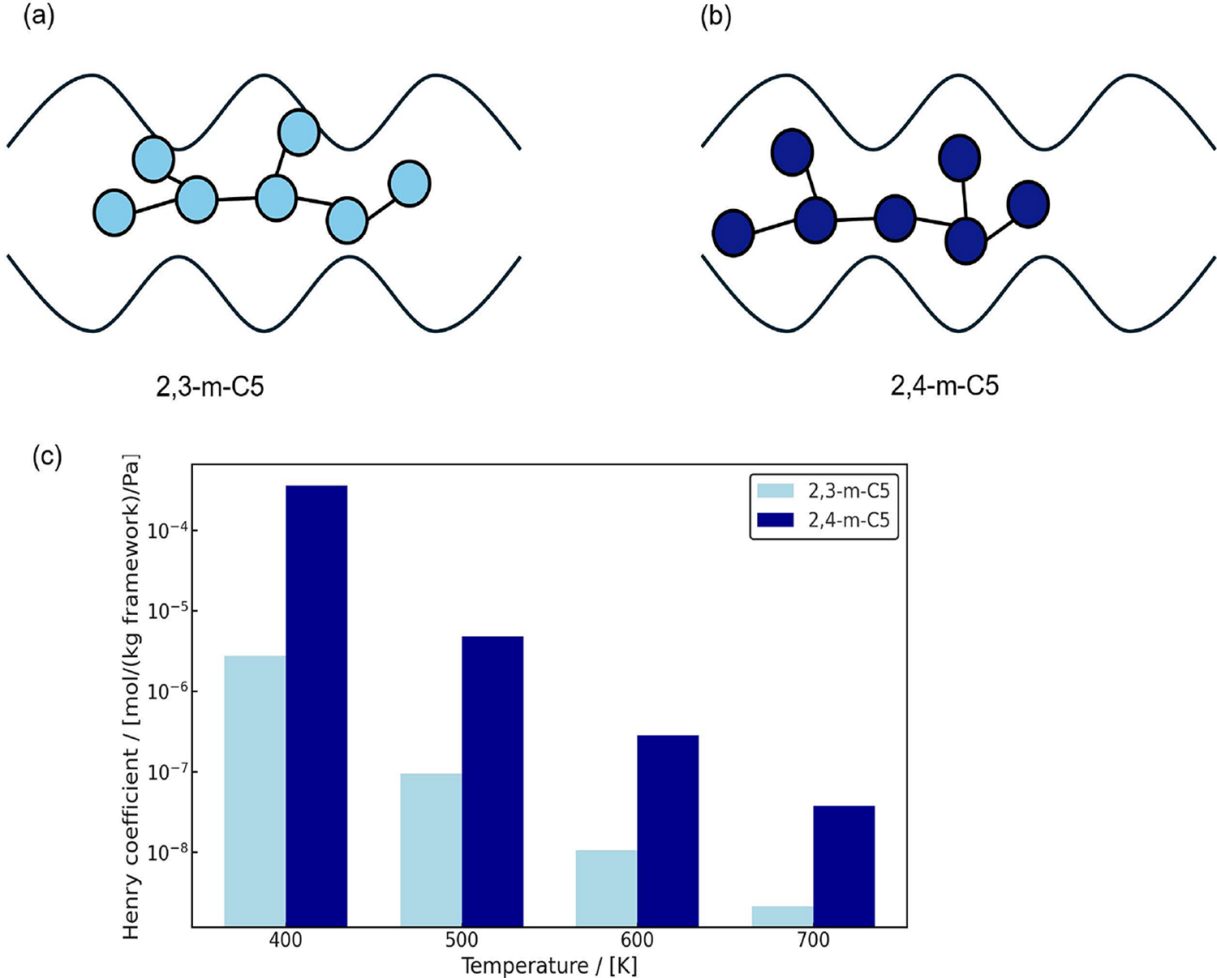
Schematic representation of the adsorption of (a) 2,3-m-C_5_ and (b) 2,4-m-C_5_ isomers in MRE-type zeolite at infinite dilution conditions. The zeolite pore corrugations arising from alternating peaks and crests^4^ induce variations in channel diameter. The larger separation between the methyl groups in 2,4-m-C_5_ enables more favorable adsorption compared to 2,3-m-C_5_. (c) Henry coefficients of 2,3-m-C_5_ and 2,4-m-C_5_ in MRE-type zeolite for the temperature range 400–700 K. Despite having structural similarities, the Henry coefficients of these isomers differ by orders of magnitude, a typical example of an activity cliff.

In this work, the performance of several ML models, including Random Forest (RF),^46^ Extreme Gradient Boosting (XGB),^47^ CatBoost (CB),^48,49^ Tabular Prior Data Fitted Network (TabPFN),^50,51^ and Directed Message Passing Neural Network (D-MPNN)^52^ are compared to predict Henry coefficients for alkane isomers in MTT-, MTW-, MRE-, and AFI-type zeolites at 523 K. RF^46^ is an ensemble learning method that constructs multiple decision trees using bootstrapped subsets of the training data and averages the predictions to improve accuracy and mitigate overfitting. It introduces additional randomness by selecting a subset of features at each split, making it highly robust to noise and capable of capturing complex feature interactions. XGB^47^ is a gradient boosting algorithm that builds trees sequentially, with each new tree learning to correct the errors of its predecessors. It incorporates advanced regularization techniques, efficient handling of missing data, and optimized tree-pruning strategies, making it exceptionally fast, scalable, and highly accurate for structured data sets. CB^48,49^ is a gradient boosting framework, specifically designed to handle categorical features natively, eliminating the need for extensive preprocessing such as one-hot encoding. It uses ordered boosting to reduce overfitting and prevent target leakage, and it constructs symmetric trees, which both accelerates training and enhances model generalization. TabPFN^50,51^ is a transformer-based model, pretrained on millions of synthetic data and fine-tuned to perform Bayesian inference on small, structured data sets. It enables near-instant, probabilistic predictions with no gradient-based training, making it especially powerful for tabular data where interpretability and uncertainty quantification are key. D-MPNN^52^ is a graph neural network architecture that represents molecules as graphs derived from the SMILES strings, where atoms are nodes and bonds are directed edges. By directed message passing, bond-level interactions are captured and encoded into molecular representations, which are subsequently processed by a feed forward neural network to predict molecular properties, offering a powerful approach for capturing intricate chemical information. For D-MPNN, the Chemprop package is used.^53–55^ For training the ML models, Henry coefficients are computed for 1110 isomers in each zeolite. The data set consists of linear (C_1_–C_30_) and branched (C_4_–C_20_) alkanes. There are 30 linear, 70 monomethyl, 435 dimethyl, and 29 trimethyl isomers, with the remainder consisting of multibranched isomers containing ethyl, methyl, propyl, and isopropyl groups. The corresponding data sets can be found in the Supporting Information SI2 folder. Both D-MPNN and TabPFN provide better predictions (i.e., exhibiting larger correlation coefficient, *R* ^2^) than the other models. In comparison to TabPFN, D-MPNN provides more accurate predictions for isomers with small Henry coefficients. The effect of active learning is analyzed to select structurally diverse isomers from the training set. This is an efficient way of exploring chemical space to identify diverse molecules for the training data set and helps in achieving better predictions with fewer training data points. Activity cliff analysis was conducted on the Henry coefficients of alkanes in MTT-type zeolites. Oversampling isomers associated with high activity cliffs in the training set led to a modest improvement in predictive performance, increasing the *R* ^2^ value by approximately 4% from 0.76 to 0.79. A more in-depth analysis is needed to achieve substantial enhancements in model accuracy. The predicted Henry coefficients, together with thermochemical properties of alkanes obtained from our previously developed linear regression model,^5^ are used to calculate the reaction equilibrium distribution of C_16_ isomers in MTW-type zeolite. The resulting distributions indicate that linear, monomethyl, dimethyl, trimethyl, tetra-methyl, and monoethyl substituted isomers are the most favored groups of isomers in this zeolite.

This article is organized as follows: Section 2 contains simulation details for Henry coefficients of alkanes in zeolites, important concepts and algorithms behind isomer enumeration, ML models, active learning, activity cliffs, and reaction equilibrium distribution. Our main results are discussed in Section 3, which includes a comparison between different ML models for predicting Henry coefficients, the use of active learning to efficiently design the training data set, the activity cliff analysis for alkanes in MTT-type zeolite, and the reaction equilibrium distribution of C_16_ isomers in one-dimensional zeolites. Section 4 provides concluding remarks on the performance of these ML models, room for future improvement for better predictive power, and their application to zeolite shape selectivity for hydroisomerization.

## 2 Methodology

Alkane isomers are generated for structures containing up to (iso)­propyl groups. We did not exclude isomers based on their potential reaction pathways. For instance, some isomers may crack rapidly and are unlikely to appear in the product distribution. Isomers with larger branches are excluded due to very unfavorable formation free energy of such alkanes in narrow-pore zeolites.^5^ The scripts to generate these isomers are provided in the Supporting Information SI1_py.py and SI1_cpp.cpp and the lists of generated isomers are available in SI1_isolist.xlsx. The algorithm for isomer generation is shown in Algorithm 1.
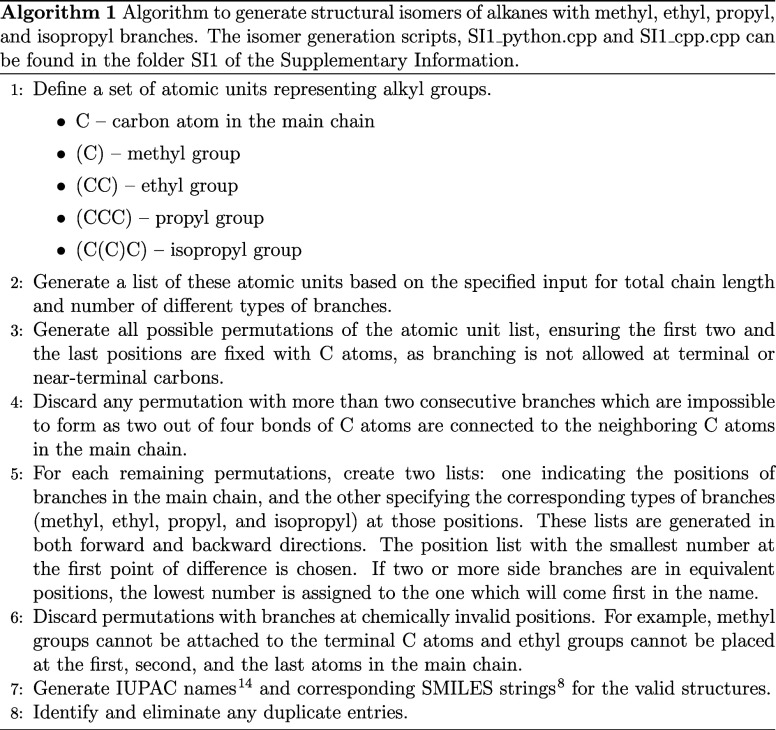


Henry coefficients for the training and testing data sets are calculated using the Widom test particle insertion method,^15^ in combination with the Configurational-Bias Monte Carlo (CBMC) technique^56,57^ using the RASPA2 software.^58–60^ In the Widom insertion test particle method, a virtual alkane molecule is randomly inserted into the zeolite simulation box at infinite dilution, and the excess chemical potential is estimated from the ensemble average of the Boltzmann factor of the Rosenbluth weight. The Henry coefficient (*k*_H_) is computed using

$$k_{H}=\frac{\lim}{P\to 0}\frac{q}{P}=\frac{1}{\rho_{\mathrm{framework}}RT}-\frac{\left\langle W_{zero}\right\rangle }{\left\langle W_{IG}\right\rangle }$$ 1

In eq 1, *q* is the amount adsorbed loadings per unit mass of the adsorbent and *P* is the pressure of the adsorbate in the fluid phase. *W*_zeo_ is the Rosenbluth weight of the alkane inside the zeolite pores and *W*_IG_ is the Rosenbluth weight of the isolated alkane molecule in the ideal gas phase, ρ_framework_ is the zeolite framework density, *R* is the universal gas constant, and *T* is the temperature. Alkanes are modeled as united-atoms,^61^ which offer a favorable balance between accuracy and computational efficiency.^62^ In this model, united atoms C, CH, CH_2_, and CH_3_ are treated as charge-neutral, allowing the omission of Coulombic interactions.^58^ The intramolecular bonded interactions are described using the TraPPE united-atom force field.^62^ Nonbonded interactions, both between adsorbent and adsorbate as well as inside adsorbate molecules, are modeled using Lennard-Jones potentials.^63^ Lennard–Jones parameters for alkanes are obtained from Dubbeldam et al.,^64^ while adsorbent–adsorbate interactions are described using the TraPPE-zeo force field.^65^ In this work, all silica zeolites are treated as rigid frameworks,^66^ since zeolite flexibility has a very small effect on adsorption behavior, particularly at infinite dilution. Lennard–Jones interactions are truncated and shifted at 12 Å without tail corrections. Cross-interactions between different atom types are handled using the Lorentz–Berthelot mixing rules.^67,68^ For each case, we performed 5,000,000 Monte Carlo cycles. In these simulations, a cycle consists of *N* steps, where *N* is the number of molecules, with a minimum of 20 steps. This ensures that, on average, one Monte Carlo move (successful or unsuccessful) is attempted for each molecule per cycle. The number of trial positions during the growth of the alkane chain is considered to be 10. Further simulation details are provided in the Supporting Information SI3.pdf. Table 2 summarizes details on the simulation box for each zeolite, such as the number of unit cells, box dimensions, and void fractions. A sufficiently large simulation box is used to eliminate finite-size effects, which is particularly important for long-chain alkanes confined in narrow zeolite pores. The Python scripts provided in ref 5 are used to automatically convert SMILES strings to input force field files for RASPA2 software.

**Table 2:** Total Number of Unit Cells for MTT-, MTW-, MRE-, and AFI-Type Zeolites Used in the Simulations to Compute the Henry Coefficients for Alkanes, Along with the Dimensions and Void Fractions of These Zeolites^a^

	number of unit cells	unit cell dimension (Å)			void fraction (−)
zeolite		*a*	*b*	*c*	
MTW-type	162 (2 × 27 × 3)	25.55	5.26	12.12	0.24
MTT-type	162 (27 × 2 × 3)	5.26	22.03	11.38	0.095
MRE-type	108 (18 × 3 × 2)	8.26	14.56	20.31	0.17
AFI-type	153 (3 × 3 × 17)	13.83	13.83	8.58	0.29

a The pore dimensions are obtained from the International Zeolite Association (IZA)^13^ and the void fractions are obtained from the iRASPA software.^33^

To predict Henry coefficients using ML, both descriptor- and graph neural network-based models are used. For descriptor-based ML models, three main types of descriptors are used: (1) total chain length, which is the total number of carbon atoms in the isomer, (2) main chain length, which is the total number of carbon atoms in the isomer excluding the branches, and (3) the number of methyl, ethyl, propyl, or isopropyl groups at each position in the main chain. These descriptors are used as input features for the RF, XGB, CB, and TabPFN models. The RF model is implemented using the Scikit-learn library,^69^ while the CB and XGB models are implemented using the CatBoost^49^ and XGBoost^47^ libraries, respectively. The D-MPNN model requires only SMILES strings as input. In this study, we used Chemprop,^53–55^ a software package for message passing neural networks, to implement the D-MPNN. The model architecture consist of a bond-based message passing network with sum aggregation, followed by a 3-layer feed-forward network. The model was trained for a maximum of 200 epochs using the Adam optimizer with a learning rate of 0.0001 and a batch size of 64. Molecular graphs are constructed using atom and bond features generated by RDKit.^70^ Atom-level features include atomic number, degree, formal charge, chiral tag, number of hydrogens, hybridization, aromaticity, and atomic mass. Bond-level features include indicators such as bond existence, bond type, conjugation, indicator of ring, and stereochemistry. As an example, the SMILES string and the descriptors for three example isomers are shown in Table 3. To ensure consistency and reproducibility, the same random splits of the data sets are used in all models. The data set is divided into training, validation, and test sets (0.72:0.08:0.2). The data in the validation set were interchanged with data from the training set to identify the best split. The test set was never part of the training.

**Table 3:** Typical Descriptors for a Few Alkane Isomers (3-e-2-m-C_6_, 4-p-C_7_, and 2-m-4-p-C_7_) Used in Training ML Models to Predict Henry Coefficients for Alkanes in Zeolites^a^

		total chain	main chain	methyl positions					ethyl positions					propyl positions				
isomer	SMILES			2	3	4	...	19	2	3	4	...	19	2	3	4	...	19
3-e-2-m-C_6_	CC(C)C(CC)CCC	9	6	1	0	0	...	0	0	1	0	...	0	0	0	0	...	0
4-p-C_7_	CCCC(CCC)CCC	10	7	0	0	0	...	0	0	0	0	...	0	0	0	1	...	0
2-m-4-p-C_7_	CC(C)CC(CCC)CCC	11	7	1	0	0	...	0	0	0	0	...	0	0	0	1	...	0

a e, m, p, and ip stands for ethyl, methyl, propyl, and isopropyl groups. Total chain (total number of C atoms in the isomer), main chain (total number of C atoms in the main chain of the isomer), and number of different types of branches at each C atom in the main chain are used for descriptor-based ML models (Random Forest, Extreme Gradient Boosting, Cat Boost, and Tabular Prior Fitted Network). SMILES strings are used as input features in Directed Message Passing Neural Network.

To evaluate the size of the training set needed to achieve satisfactory accuracy in predicting Henry coefficients for a diverse range of alkane isomers in zeolites, we compared a random selection strategy and an active learning strategy^71^ for building successively larger training data sets. The active learning algorithm uses Gaussian process regression with a marginalized graph kernel (GPR-MGK)^71^ as the surrogate model. At each iteration, a GPR-MGK model is constructed from the current training set to estimate the predictive uncertainties of the remaining molecules, and the molecule with the largest uncertainty is added to the training set until 50 new isomers are identified. To provide some intuition about the selections by the active learning algorithm, the initial 50 molecular structures selected by active learning from the training sets, comprising linear alkanes (C_1_–C_30_) and methyl-branched alkanes (C_4_–C_20_), as well as linear alkanes (C_1_–C_30_) and methyl-, ethyl-, propyl-, and isopropyl-branched alkanes (C_4_–C_20_) are shown in Figure S10 a,b in the Supporting Information SI3.pdf. Following each active learning iteration, the D-MPNN and the TabPFN models were retrained on the expanded training set. To ensure robust training and prevent overfitting across the different data set sizes, the D-MPNN model was trained with a consistent set of hyperparameters. We employed a dropout rate of 0.2 in the 3-layer feed-forward network and an early stopping protocol with a patience of 25 epochs based on the validation loss. Furthermore, a batch size of 50 was used to ensure stable gradient updates and prevent instabilities arising from uneven final batches in the dataloader. The model performance at each iteration was evaluated using the same full validation and test sets as described above.

The Structure Activity Landscape Index (SALI) is used to quantify activity cliffs in isomers for Henry coefficients. This index is a pairwise score that captures the magnitude of the property change with respect to the distance of two compounds in the chemical space.^34^

$$\mathrm{SALI}=\frac{\left|-\ln(k_{H,i})-(-\ln(k_{H,j}))\right|}{d_{i,j}}$$ 2

In eq 2, *d*_*i*,*j*_ is the structural distance between a pair of isomers *i* and *j*. In this work, Levenshtein distance^39^ between SMILES strings is used as *d*_*i*,*j*_. This distance quantifies dissimilarities between two strings by counting the minimum number of single character edits, which include insertions, deletions, or substitutions, required to transform one string into the other. Pairwise activity cliffs measured using SALI are computed for the Henry coefficients of alkane isomers adsorbed in MTT-type zeolites at 523 K. Based on the SALI values, the data set is divided into low and high activity cliff subsets. 70% of the low cliff data and 20% of the high cliff data are added to the training set randomly. Oversampling is performed by duplicating the high-cliff data. A comparison is made between the predictions of TabPFN models with and without oversampling of high activity cliff data. The detailed algorithm is presented in Algorithm 2.
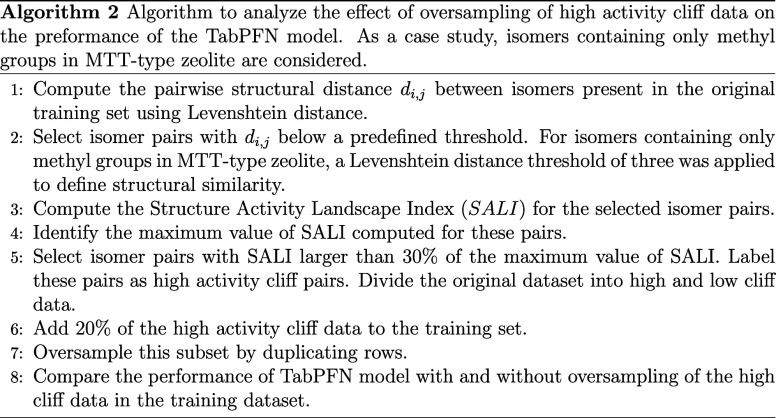


The predicted Henry coefficients for C_16_ isomers at 523 K are used to compute the reaction equilibrium distribution for hydroisomerization of linear C_16_ into branched isomers at infinite dilution in MTW-, MTT-, MRE-, and AFI-type zeolites. The chemical reaction equilibrium distribution is obtained by imposing a gas phase reaction equilibrium for the alkane isomers and a simultaneous phase equilibrium between the gas and the adsorbed phase for each component.^4,5^ This satisfies the reaction equilibrium distribution in the zeolites,^72^ which is typically valid at low conversions of hydrocarbons. The adsorbed phase loadings for these isomers are modeled using Henry’s law. The Henry coefficients are predicted using the ML models. Henry coefficients are predicted using the D-MPNN model for MTT-, and MRE-type zeolites, while the TabPFN model is used for the MTW- and AFI-type zeolites. For further details on this method, the reader is referred to refs 4 and 5. The gas phase reaction equilibrium distribution is computed using the Gibbs free energy (*G*_0_(*T*) – *H*_0_(0 K)) at the operating temperature *T* (523 K) and enthalpy of formation (Δ_f_ *H*_0_(0 K)) at 0 K.^4^ These properties are predicted using our linear regression model^5^ based on a second-order group contribution method. The reaction equilibrium distribution is characterized by the selectivities of the isomers relative to the linear alkane in the adsorbed phase. Absolute selectivities are defined as the ratio of the mole fraction of a component to the sum of the mole fractions of all other components in the adsorbed phase.^73^ Relative selectivities compare the absolute selectivity of a specific isomer to that of the corresponding linear alkane, providing a measure of preferential adsorption. For further details, the reader is referred to eqs 21–24 in ref 4.

## 3 Results and Discussion

Figure 3a shows coefficients of determination, *R* ^2^ of D-MPNN, RF, XGB, CB, and TabPFN for predicting the negative logarithm of Henry coefficients, −ln­(*k*_H_), for linear (C_1_–C_30_) and methyl-branched (C_4_–C_20_) alkanes in MTT-, MTW-, MRE-, and AFI-type zeolites at 523 K. Error bars are small for most alkane isomers, indicating reliable estimates of the Henry coefficients. Small error bars ensure that the training data supplied to ML models is accurate and consistent, thereby improving model generalization. For highly branched isomers, the error bars can be relatively large compared to the Henry coefficients, due to poor fit inside the zeolite pores and challenges in achieving convergence in Monte Carlo simulations. These highly branched isomers are of limited practical relevance, as these isomers occur only in negligible amounts in the product distribution of hydroisomerization reactions. Since operating pressures are at most 20 bar, Henry coefficients on the order of 10^–40^ or 10^–80^ will both lead to virtually zero adsorption, and thus the exact value is irrelevant. Further details on the error bars are provided in the Supporting Information SI3.pdf. −ln­(*k*_H_) is chosen instead of *k*_H_ because Henry coefficients of alkanes vary orders of magnitude. ML models perform better on an evenly distributed data set where the target variable has a Gaussian-like distribution.^74^ This can be achieved using −ln­(*k*_H_) instead of *k*_H_. D-MPNN and TabPFN perform better than the RF, XGB, and CB models. In AFI-type zeolite, all ML models achieve high accuracy with *R* ^2^ values exceeding ca. 0.98. TabPFN outperforms other models for MTW-type zeolite with 0.98 *R* ^2^, and D-MPNN delivers superior performance in MTT- and MRE-type zeolites with *R* ^2^ values of 0.95 and 0.96 respectively. Figure 3b,c shows the parity plots predicted by TabPFN and D-MPNN for linear (C_1_–C_30_) and methyl-branched alkanes (C_4_–C_20_) in MTW-type zeolite at 523 K. These plots provide predictions for isomers present in the training (blue circles), validation (green circles), and test (red circles) sets. For alkanes larger than C_20_, predictions by both TabPFN and D-MPNN deviate from the actual values due to lack of training data in this range. D-MPNN performs better than TabPFN for low Henry coefficients or high values for −ln­(*k*_H_). These are usually highly branched isomers. For applications such as hydroisomerization, highly branched isomers will not form inside the narrow pores of one-dimensional zeolites such as MTW-, MTT-, and MRE-type zeolites.^4,5^ The parity plots for MTT-, MRE-, and AFI-type zeolites are included in the Supporting Information SI3.pdf.

**Figure 3:**
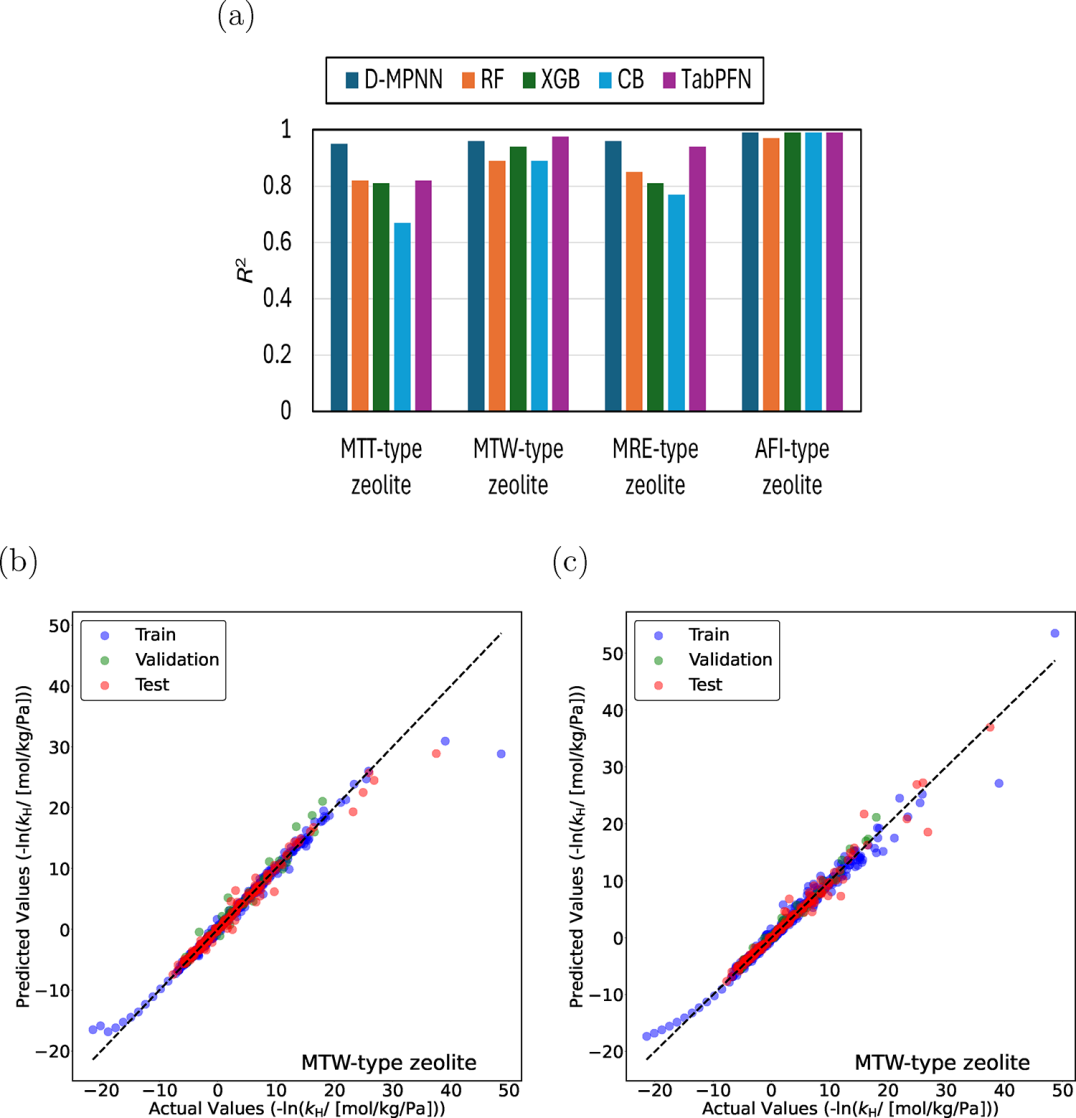
(a) Coefficients of determination (*R* ^2^) for Directed Message Passing Neural Network (D-MPNN), Random Forest (RF), Extreme Gradient Boosting (XGB), CatBoost (CB), and Tabular Prior Fitted Network (TabPFN) models predicting the negative logarithm of Henry coefficients, −ln­(*k*_H_), for linear alkanes (C_1_–C_30_) and methyl-branched alkanes (C_4_–C_20_) in MTT-, MTW-, MRE-, and AFI-type zeolites at 523 K. The unit of *k*_H_ is mol/kg framework/Pa. Models are trained separately for each zeolite. Parity plots for predictions of −ln­(*k*_H_) in MTW-type zeolite at 523 K using (b) TabPFN and (c) D-MPNN models. Blue circles indicate training isomers, and green circles represent validation isomers. The random seed is varied to identify the most suitable split between the training and the validation data sets. Red circles represent the test isomers, which are never part of the training set. The standard deviations of the actual values of −ln­(*k*_H_) are less than 10% of the actual values.

Figure 4a shows the coefficient of determination, *R* ^2^ for D-MPNN, RF, XGB, CB, and TabPFN for predicting −ln­(*k*_H_) for linear (C_1_–C_30_) and methyl-, ethyl-, propyl-, and isopropyl-branched (C_4_–C_20_) alkanes in MTT-, MTW-, MRE-, and AFI-type zeolites at 523 K. For MTT-type zeolite, XGB performs slightly better (larger *R* ^2^) than the other ML models. For MTW- and AFI-type zeolites, TabPFN provides better predictions with *R* ^2^ values of ca. 0.96 and 0.94, respectively. D-MPNN performs better for MRE-type zeolite with *R* ^2^ ca. 0.95. Figure 4b,c shows the parity plots for −ln­(*k*_H_) predicted by TabPFN and D-MPNN for linear (C_1_–C_30_) and methyl-, ethyl-, propyl-, and isopropyl-branched alkanes (C_4_–C_20_) in MTW-type zeolite. Similar to the data set with all methyl groups, D-MPNN performs better at low Henry coefficients compared to TabPFN when alkanes with ethyl, propyl, and isopropyl branches are introduced to the data set. Each model provides reasonable predictions. It is difficult to claim a single best model for predicting Henry coefficients. TabPFN and D-MPNN have shown more consistency in predictions compared to the other tree-based models (Figures 3a and 4a). The parity plots for monomethyl and dimethyl alkanes obtained using the TabPFN model are shown in Figures 5 and 6. TabPFN provides excellent predictions for mono- and dimethyl alkanes in MTW-, MTT-, MRE-, and AFI-type zeolites (*R* ^2^ > 0.92). To further improve the predictability of the models, a larger data set is required, and structurally diverse isomers need to be included in the training set using advanced methods such as active learning.

**Figure 4:**
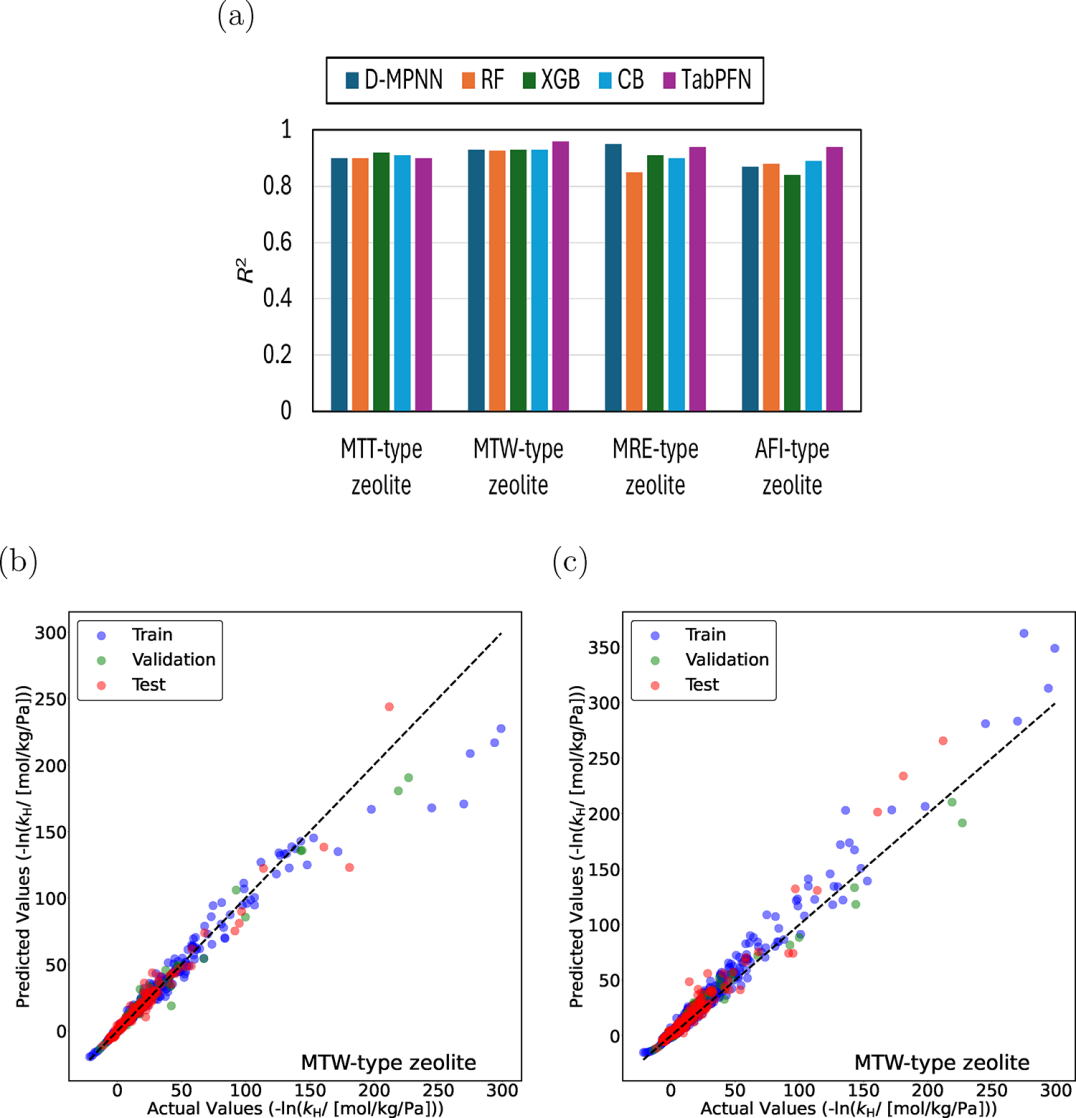
(a) Coefficients of determination, *R* ^2^ for Directed Message Passing Neural Network (D-MPNN), Random Forest (RF), Extreme Gradient Boosting (XGB), CatBoost (CB), and Tabular Prior Fitted Network (TabPFN) models predicting the negative logarithm of Henry coefficients, −ln­(*k*_H_), for linear alkanes (C_1_–C_30_) and methyl-, ethyl-, propyl-, and isopropyl-branched alkanes (C_4_–C_20_) in MTT-, MTW-, MRE-, and AFI-type zeolites at 523 K. ML models are trained separately for each zeolite. Parity plots for −ln­(*k*_H_) predictions in MTW-type zeolite at 523 K using (b) TabPFN and (c) D-MPNN models. Blue circles indicate training isomers and green circles represent validation isomers. The random seed is varied to identify the most suitable split between the training and the validation data sets. Red circles represent the test isomers, which are never part of the training set. The standard deviations for the actual values of −ln­(*k*_H_) are too small to plot.

**Figure 5:**
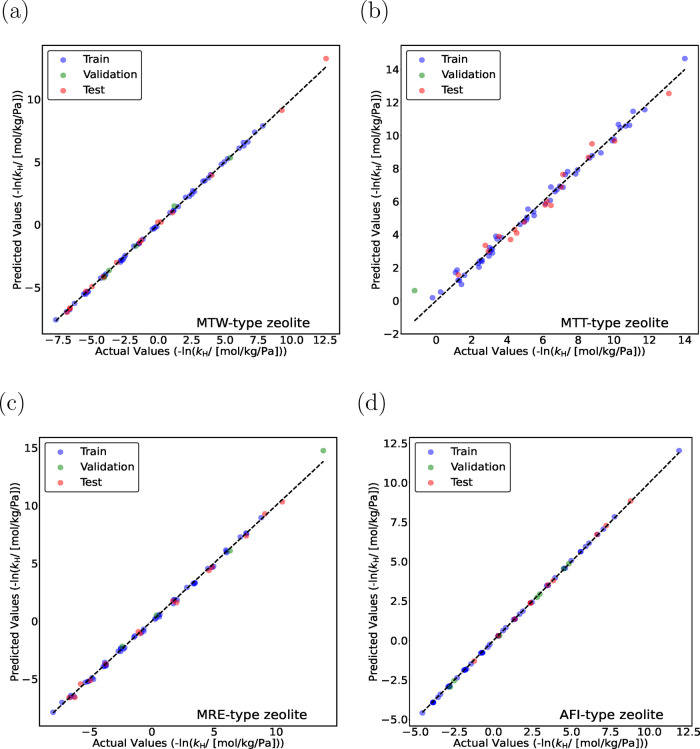
Parity plots for predicting −ln­(*k*_H_) for monomethyl alkanes (C_4_–C_20_) in (a) MTW-, (b) MTT-, (c) MRE-, and (d) AFI-type zeolites at 523 K using the TabPFN model. The training data sets contain linear (C_1_–C_30_) and methyl-, ethyl-, propyl-, and isopropyl-branched (C_4_–C_20_) alkanes. Blue circles indicate training isomers, and green circles represent validation isomers. The random seed is varied to identify the most suitable split between the training and the validation data sets. Red circles represent the test isomers, which are never part of the training set. The standard deviations for the actual values of −ln­(*k*_H_) are too small to plot.

**Figure 6:**
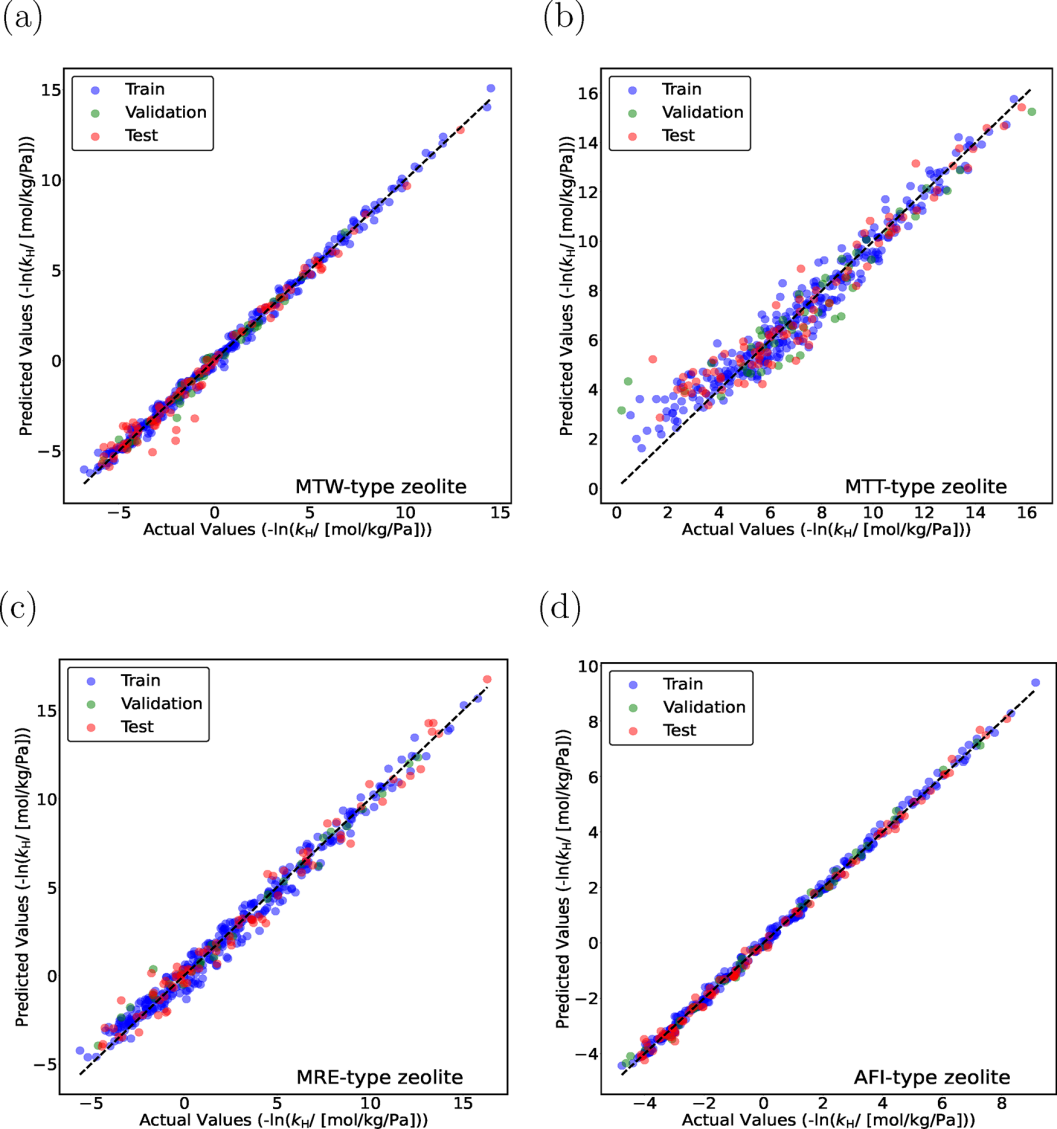
Parity plots for predicting −ln­(*k*_H_), for dimethyl alkanes (C_5_–C_20_) in (a) MTW-, (b) MTT-, (c) MRE-, and (d) AFI-type zeolites at 523 K using the TabPFN model. The training data sets contain linear (C_1_–C_30_) and methyl-, ethyl-, propyl-, and isopropyl-branched (C_4_–C_20_) alkanes. Blue circles indicate training isomers, and green circles represent validation isomers. The random seed is varied to identify the most suitable split between the training and the validation data sets. Red circles represent the test isomers, which are never part of the training set. The standard deviations for the actual values of −ln­(*k*_H_) are too small to plot.

In Figures 7 and 8, the influence of training set size on the predictive accuracy of the TabPFN and D-MPNN models for −ln­(*k*_H_) is shown for linear (C_1_–C_30_) and branched (methyl-, ethyl-, propyl-, and isopropyl-, C_4_–C_20_) alkanes in MTW- and MTT-type zeolites at 523 K, comparing active learning and random selection strategies. For the TabPFN model (Figure 7), active learning consistently resulted in higher predictive accuracy for both zeolite types. In case of MTW, model performance stabilized beyond 250 training points, with *R* ^2^ reaching ca. 0.975, whereas random selection exhibited substantial fluctuations, yielding *R* ^2^ values of only ca. 0.88 even at 500 training points (Figure 7a,b). A similar trend was observed for MTT, where active learning achieved stable predictions of −ln­(*k*_H_) beyond 250 training points, with *R* ^2^ of ca. 0.92 and RMSE of ca. 10, clearly outperforming random selection (Figure 7c,d). In contrast, a random selection of 600 training points in MTT yielded an *R* ^2^ of only 0.67. For the D-MPNN model (Figure 8), the primary advantage of active learning was the increased stability and consistency of the training process. While the mean performance of random selection occasionally caught or exceeded that of active learning at certain data sizes due to statistical chance, its performance was unreliable, characterized by large fluctuations and high variance (indicated by the large error bars in Figure 8 especially 650 data set size for MTT-type zeolite). In contrast, the active learning provided a more monotonic and reliable improvement, having either comparable performance in the larger data set size or better performance in the smaller data set size. For example, in the MTW-type zeolite (Figure 8a,b), active learning steadily converged to a stable *R* ^2^ of 0.95, whereas the random selection strategy remained erratic throughout the process. A direct comparison between the two models reveals a key difference in their data efficiency. The TabPFN model demonstrated remarkable performance even with very small training sets. For instance, in the MTW zeolite, TabPFN achieved an *R* ^2^ of approximately 0.95 with only 150 training points (Figure 7a). In contrast, the D-MPNN model required around 350 data points to stably reach a similar level of accuracy (Figure 8a). This trend suggests that while both models can ultimately achieve high predictive accuracy, TabPFN is significantly more data-efficient, making it a strong candidate for scenarios where labeled data is particularly scarce. This higher data efficiency can be partly attributed to the strong prior knowledge encoded in TabPFN’s Transformer architecture.^51^ Overall, for both the TabPFN and the D-MPNN models, the active learning strategy delivered a more systematic and robust path to high performance. It was characterized by lower variance and a more reliable improvement in *R* ^2^ and RMSE values, making it a superior strategy for efficiently training models with limited labeled data compared to the less reliable, high-variance outcomes of random selection. A notable exception to this trend occurs at the smallest training set size of 50 for the D-MPNN model, where random selection exhibits a higher initial accuracy. This phenomenon can also be found for models trained on linear (C_1_–C_30_) and methyl-branched (C_4_–C_20_) alkanes, as shown in Figure S9 in the Supporting Information SI3.pdf. This is a known phenomenon in active learning referred to as the cold start problem.^75^ At this early stage, the active learning strategy prioritizes exploration by selecting diverse, high-uncertainty samples to efficiently map the feature space. While crucial for long-term performance, these samples can be initially challenging for the neural networks to learn. Conversely, random selection can, by chance, sample a more homogeneous cluster of simpler alkanes, leading to a deceptively strong initial performance.

**Figure 7:**
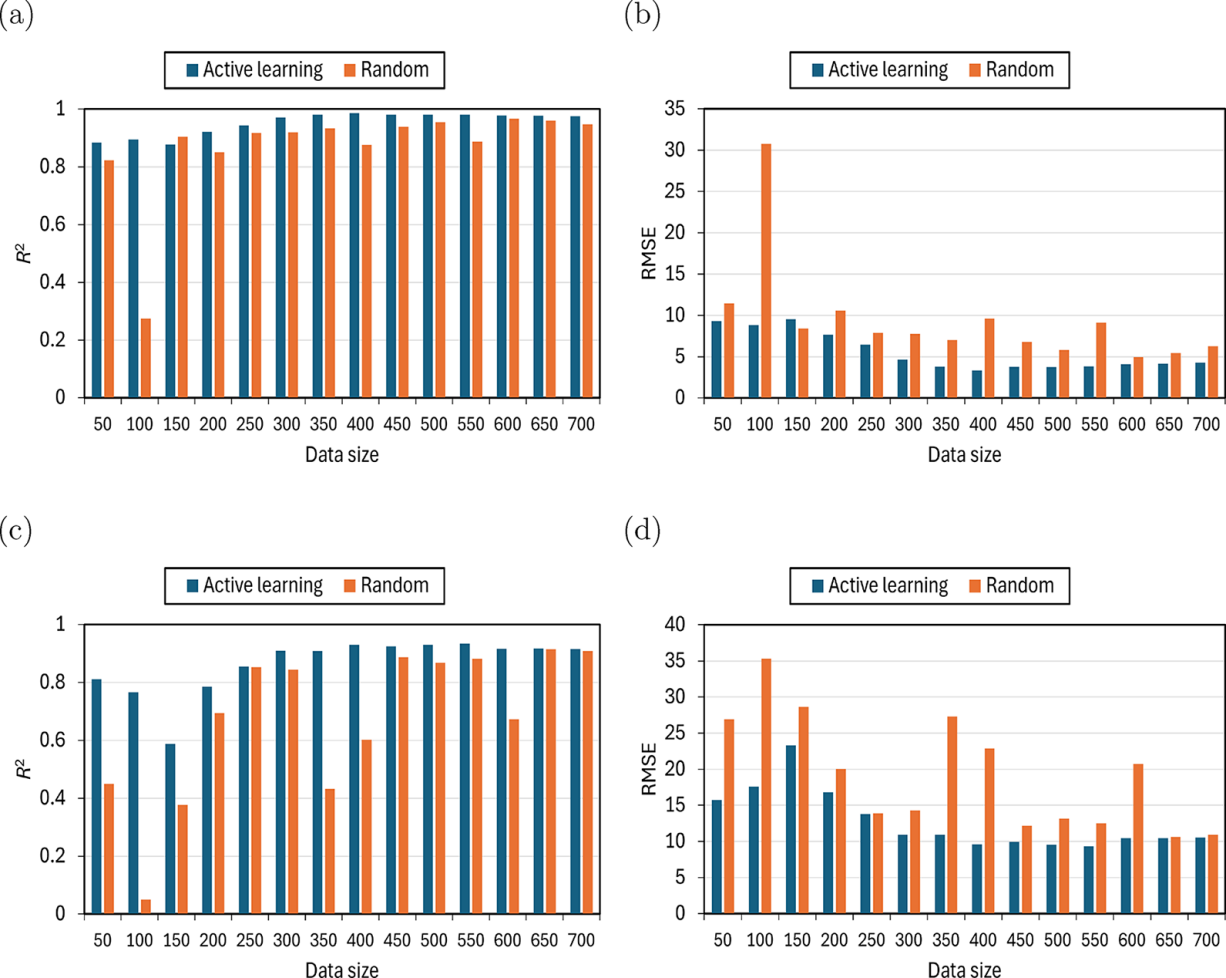
Test accuracies of the TabPFN model as a function of training set size, comparing an active learning strategy (blue bars) and a random selection strategy (orange bars). *R* ^2^ and RMSE values are shown for models trained on the negative logarithm of Henry coefficients for linear (C_1_–C_30_) and methyl-, ethyl-, propyl-, and isopropyl-branched (C_4_–C_20_) alkanes in MTW-type (a, b) and MTT-type (c, d) zeolites at 523 K.

**Figure 8:**
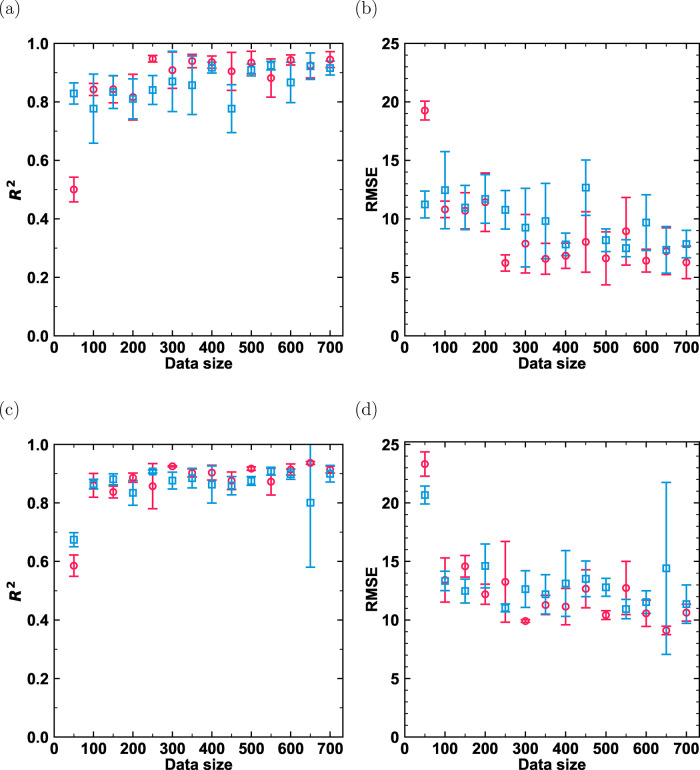
Test accuracies of the D-MPNN model as a function of training set size, comparing an active learning strategy (red circles) and a random selection strategy (blue squares). *R* ^2^ and RMSE values are shown for models trained on the negative logarithm of Henry coefficients for linear (C_1_–C_30_) and methyl-, ethyl-, propyl-, and isopropyl-branched (C_4_–C_20_) alkanes in MTW-type (a, b) and MTT-type (c, d) zeolites at 523 K.

Figure 9a,b shows parity plots for −ln­(*k*_H_) predicted by TabPFN and D-MPNN for linear (C_1_–C_30_) and methyl-branched alkanes (C_4_–C_20_) in MTT-type zeolites. TabPFN exhibits larger deviations in the low Henry coefficient regime compared to D-MPNN, especially for highly branched isomers and those with closely spaced branching points. A major contributing factor to this discrepancy is the limited representation of highly branched alkanes in the training data set, which reduces the ability of the ML algorithms to learn accurate structure–property relationships in these regions. Additionally, pronounced activity cliffs can be observed for such isomers, where minor structural changes lead to substantial variations in adsorption behavior (Table 4). Such cliffs violate the similarity assumption underpinning many machine learning and QSAR models, namely that structurally similar molecules should exhibit similar properties.^35^ Table 5 shows the effect of oversampling high-cliff isomers^34^ on TabPFN predictions for MTT-type zeolites, using three different train-test splits. In each case, 20% of the high-cliff isomers were included in the training set. Oversampling led to a modest improvement in accuracy, with an average *R* ^2^ increase of approximately 4%. Improvements in predictions of *k*_H_ for isomers due to oversampling are shown in Table S7 in the Supporting Information SI3.pdf. Additional data points and strategies such as contrastive learning^76,77^ may be necessary to better capture sharp structure–property discontinuities. Furthermore, the Henry coefficients of these isomers may be sensitive to the treatment of framework flexibility, adding further complexity to prediction efforts.

**Table 4:** Pronounced Activity Cliffs in Henry Coefficients for Alkane Isomer Pairs with Small Variations in Branching Positions in MTT-Type Zeolite at 523 K

isomer	Henry coefficient (mol/kg/Pa)	Isomer	Henry coefficient (mol/kg/Pa)
5,5-m-C_10_	1.54 × 10^–4^	5,6-m-C_10_	3.42 × 10^–6^
4,4-m-C_14_	1.38 × 10^–3^	4,5-m-C_14_	8.66 × 10^–5^
2,3,3-m-C_6_	7.73 × 10^–10^	2,3,5-m-C_6_	1.51 × 10^–7^

**Figure 9:**
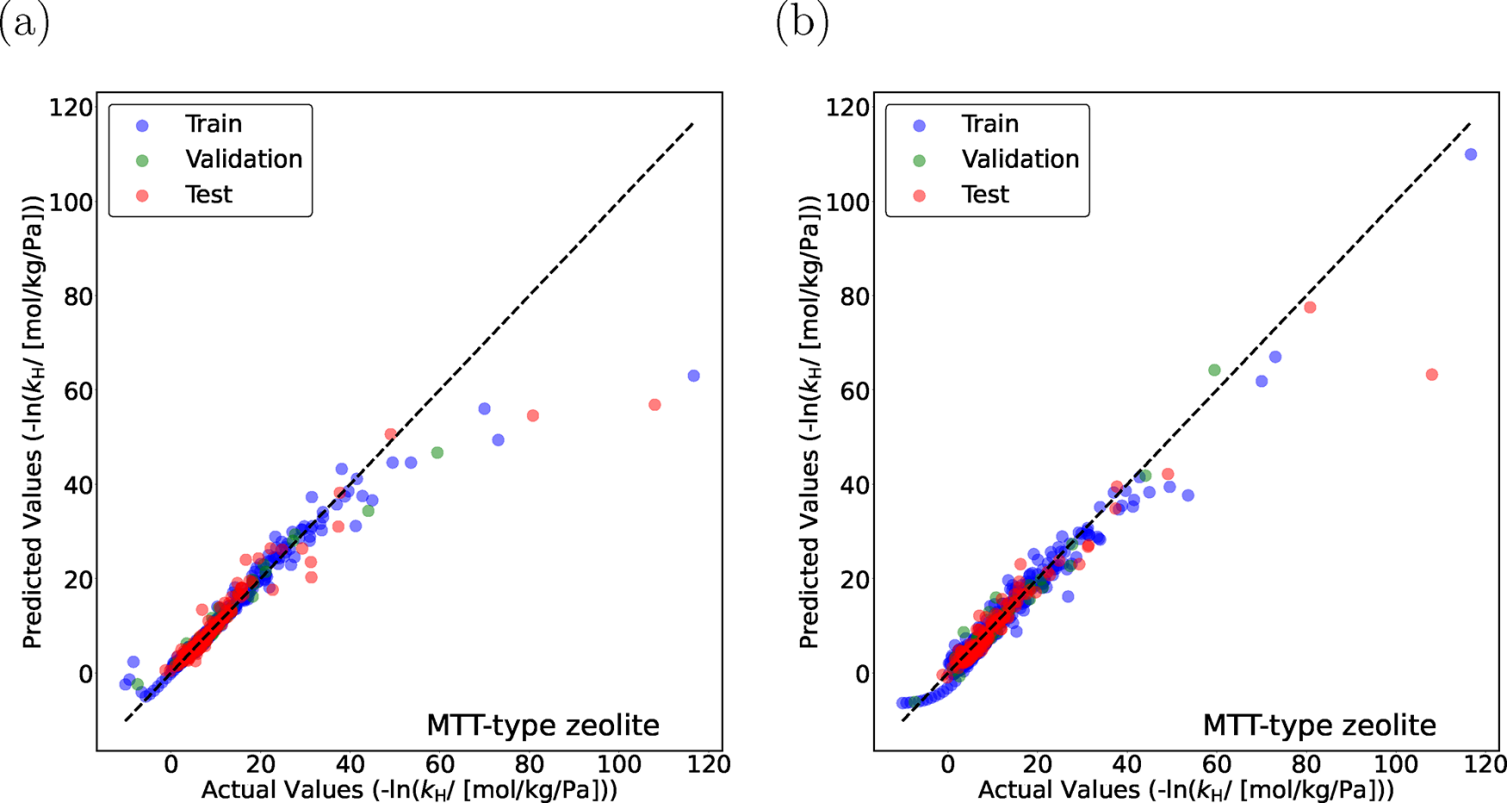
Parity plots for the negative logarithm of Henry coefficients, −ln­(*k*_H_) for linear alkanes (C_1_–C_30_) and methyl-branched alkanes (C_4_–C_20_) in MTT-type zeolite at 523 K predicted by (a) Tabular Prior Fitted Network (TabPFN) and (b) Directed Message Passing Neural Network (D-MPNN) models. Blue circles indicate training isomers, and green circles represent validation isomers. The random seed is varied to identify the most suitable split between the training and the validation data sets. Red circles represent the test isomers, which are never part of the training set. The standard deviations for the actual values of −ln­(*k*_H_) are too small to plot.

**Table 5:** Effect of Oversampling 20% High Activity Cliff Data on the Prediction Accuracy (*R* ^2^) of TabPFN for Methyl-Branched and Linear Alkanes in MTT-Type Zeolite at 523 K at Different Random States, Representing Different Train-Test Splits and the Corresponding Average *R* ^2^ ^a^

	*R* ^2^ at different random states				% increase in *R* ^2^
data set	0	42	90	average	
without oversampling	0.73	0.77	0.78	0.76	3.74
with oversampling	0.75	0.82	0.80	0.79	

a There is an average 3.74% increase in *R* ^2^ by oversampling 20% high activity cliff data.

Figure 10a shows the variations in average Henry coefficients for different groups of C_16_ isomers in MTW-type zeolite at 523 K where the coefficients are averaged in each category. As a narrow-pore zeolite, MTW-type zeolite^13^ preferentially adsorbs linear C_16_, followed by isomers with mono-, di-, tri-, and tetra-methyl groups, as well as monoethyl substitutions. In sharp contrast, highly branched isomers, particularly those containing both monoethyl and mono­(iso)­propyl groups, exhibit significantly smaller adsorption affinity. A similar trend is observed in the relative selectivities of C_16_ isomers for hydroisomerization at reaction equilibrium (Figure 10b). In MTW, the adsorption strength, as reflected by the Henry coefficients, plays a more dominant role than the gas-phase thermochemical properties in determining the reaction equilibrium distribution. In MTT-, MRE-, and AFI-type zeolites, isomers with mono-, di-, tri-, and tetra-methyl branching are also the most favored groups in terms of relative selectivity for hydroisomerization of linear C_16_ at reaction equilibrium. Bar plots summarizing the average Henry coefficients and relative selectivities for C_16_ isomer groups in these zeolites are provided in SI3.pdf. The reaction equilibrium distribution of linear, monomethyl (2-m-C_15_ to 8-m-C_15_) and dimethyl (2,2-m-C_14_ to 2,13-m-C_14_) isomers of C_16_ are shown in Figure S11 in SI3.pdf. Accurate prediction of Henry coefficients is essential for reliable computation of reaction equilibrium distributions. Therefore, a high coefficient of determination, *R* ^2^ serves as a critical performance metric for model evaluation. This work will be further extended to enhance the predictive performance of the ML models by incorporating larger data sets and using advanced techniques such as active learning that include a more diverse set of alkane isomers in the training set. Additionally, a generalized ML framework will be developed which will be capable of predicting Henry coefficients for various alkanes in different types of zeolite structures.

**Figure 10:**
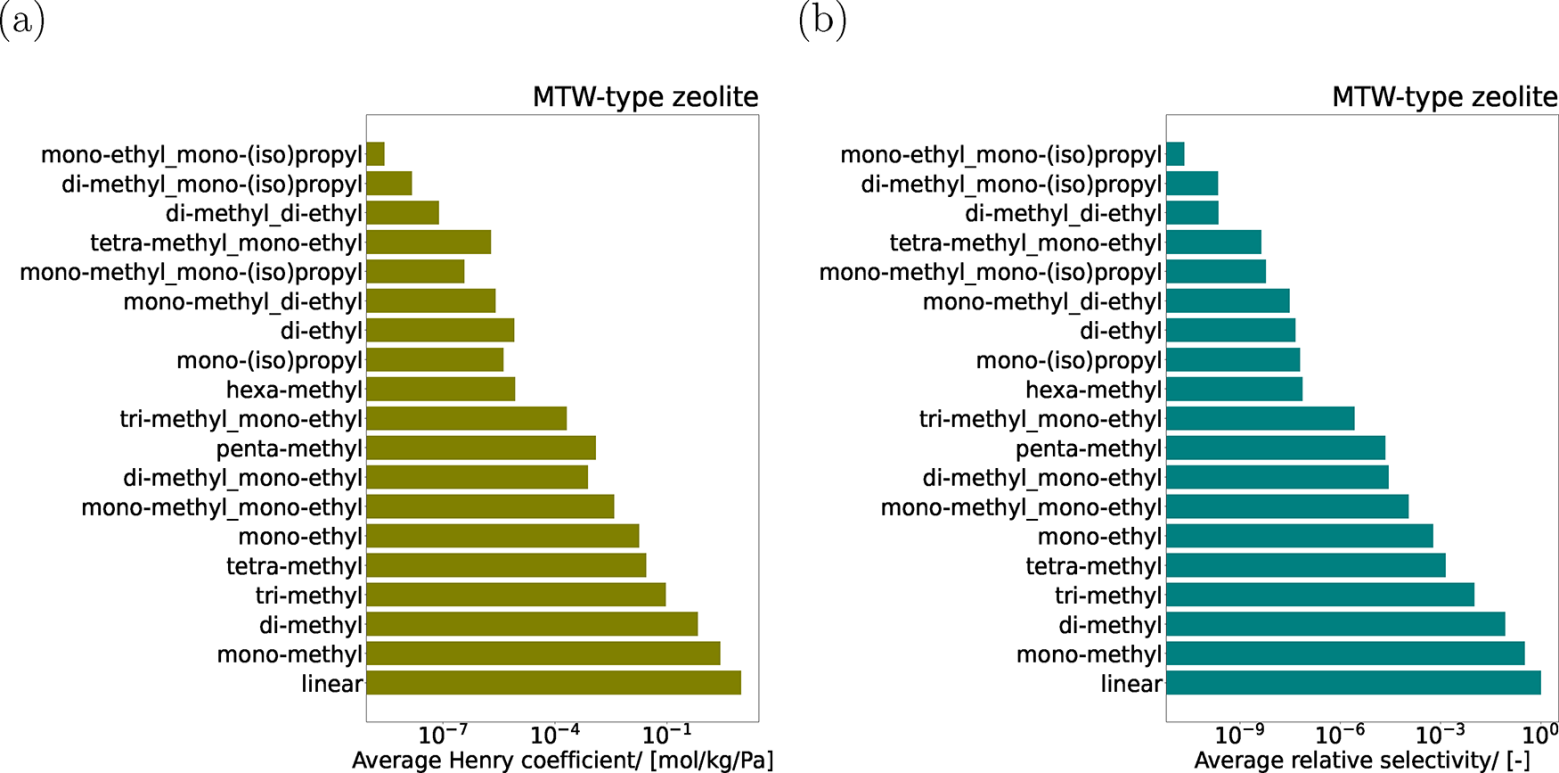
(a) Average Henry coefficients for different categories of C_16_ isomers in MTW-type zeolite at 523 K predicted using the TabPFN model. For each category, the Henry coefficients are averaged over all the isomers belonging to that category. (b) Average selectivities of different categories of C_16_ isomers relative to linear C_16_ at reaction equilibrium in MTW-type zeolite at 523 K. For monomethyl isomers, 2-m-C_15_ has the largest Henry coefficient and the relative selectivity. Similarly, 2,13-m-C_14_ and 2,6,10-m-C_13_ have the largest selectivities for di- and trimethyl isomers, respectively.

## 4 Conclusions

This study presents an ML framework for predicting Henry coefficients of long-chain alkanes in one-dimensional zeolites using both descriptor- and graph-based models, as well as software for the fast enumeration of hydrocarbon isomers. For the evaluated models, TabPFN and D-MPNN demonstrate consistently excellent predictive performance, particularly for linear and moderately branched isomers. TabPFN provides excellent predictions for mono- and dimethyl alkanes in MTW-, MTT-, MRE-, and AFI-type zeolites. D-MPNN exhibits better accuracy for isomers with low Henry coefficients, which are typically highly branched and difficult to adsorb in narrow-pore zeolites. Accurately predicting Henry coefficients for highly branched isomers remains challenging, primarily due to the limited representation of diverse branching patterns in the training data set. The presence of pronounced activity cliffs, where small structural changes can lead to order of magnitude differences in values further increase the need for additional data to adequately capture such effects. Active learning strategies and oversampling of high-cliff data led to modest improvements in model accuracy, indicating the potential of these techniques for future development. Underprediction of adsorption for bulky isomers may not significantly impact practical applications such as hydroisomerization, since such isomers are sterically excluded from narrow zeolite pores. The predicted Henry coefficients, combined with gas-phase thermodynamic properties derived from a second-order group contribution model, enable the computation of reaction equilibrium distributions for hydroisomerization processes. This integrated approach provides valuable insights into the thermodynamic versus kinetic-driven selectivity of isomers, aiding the development of lumped kinetic models and catalyst design. Future work will focus on expanding the data set by incorporating more chemically diverse isomers, including those with ethyl, propyl, and isopropyl branches, using active learning and other advanced sampling techniques. The development of a generalizable ML framework for predicting Henry coefficients of a broad range of alkanes in different zeolite topologies is envisioned. This will facilitate high-throughput screening and rational catalyst design for hydrocarbon upgrading applications.

## Supplementary Material





## Notes

Isomer generation scripts (SI1_python.py and SI1_cpp.cpp) and an excel file SI1_isolist.xlsx containing all alkane isomers ranging from C_1_ to C_20_; the ML scripts, SI2_Descriptor_based.py for descriptor based models, RF, XGB, CB, and TabPFN and SI2_DMPNN.py for D-MPNN model; SI2 folder also contains SI2_m-Cn_MTT.txt, SI2_m-Cn_MTW.txt, SI2_m-Cn_MRE.txt, SI2_m-Cn_AFI.txt, SI2_m-e-p-ip-Cn_MTT.txt, SI2_m-e-p-ip-Cn_MTW.txt, SI2_m-e-p-ip-Cn_MRE.txt, and SI2_m-e-p-ip-Cn_AFI.txt; data sets are used for training, validation, and testing data for descriptor based models; files are named according to the types of isomers and the zeolite, for example, SI2_m-Cn_MTT.txt contains data for linear alkanes and isomers with methyl branches in MTT-type zeolite; SI2_m-e-p-ip-Cn_MTW.txt contains linear alkanes and isomers with methyl, ethyl, propyl, and isopropyl branches in MTW-type zeolite; SI2 folder also contains data sets in *.csv* format which includes SI2_m-Cn_MTT.csv, SI2_m-Cn_MTW.csv, SI2_m-Cn_MRE.csv, SI2_m-Cn_AFI.csv, SI2_m-e-p-ip-Cn_MTT.csv, SI2_m-e-p-ip-Cn_MTW.csv, SI2_m-e-p-ip-Cn_MRE.csv, SI2_m-e-p-ip-Cn_AFI.csv; data sets are used for the D-MPNN model; Henry coefficients computed using molecular simulations are provided in SI2_HC.xlsx; and the reaction equilibrium distributions for C_16_ isomers, computed using the Henry coefficients predicted by our ML framework, also included in SI2_HC.xlsx (ZIP)
Parity plots comparing the predicted Henry coefficients with the actual Henry coefficients for alkanes in MTT-, MTW-, MRE-, and AFI-type zeolites; bar plots showing the Henry coefficients averaged over the isomers in each category of C_16_ isomers in MTW-, MTT-, MRE-, and AFI-type zeolites; it presents the average selectivities for hydroisomerization, computed for the isomers in each group for the same zeolites; and the initial 50 molecular structures selected by active learning (PDF)
