# Supplementary material for: Machine Learning-Based Predictions of Henry Coefficients for Long-Chain Alkanes in One-Dimensional Zeolites: Application to Hydroisomerization
Source: J Phys Chem C Nanomater Interfaces. 2025 Sep 26;129(40):18234–49. doi: 10.1021/acs.jpcc.5c03868 (PMC12516733; doi:10.1021/acs.jpcc.5c03868)
Supplement: Supplementary file 2 [file jp5c03868_si_002.pdf]

Supporting Information: Machine Learning-Based Predictions of Henry Coefficients for Long Chain Alkanes in One Dimensional Zeolites: Application to Hydroisomerization

Shrinjay Sharma,[†,‡,¶] Ping Yang,[§,¶] Yachan Liu,[§] Kevin Rossi,[‖,⊥] Peng Bai,[§] Marcello S. Rigutto,[#] Erik Zuidema,[#] Umang Agarwal,[@] Richard Baur,[#] Sofia Calero,[‡] David Dubbeldam,[△] and Thijs J.H. Vlugt[*,†]

†*Process & Energy Department, Faculty of Mechanical Engineering, Delft University of Technology, Leeghwaterstraat 39, 2628CB Delft, The Netherlands*

‡*Department of Applied Physics and Science Education, Eindhoven University of Technology, 5600MB Eindhoven, The Netherlands*

¶*These authors contributed equally to this work.*

§*Department of Chemical Engineering, University of Massachusetts Amherst, Amherst, MA 01003, United States*

‖*Department of Materials Science and Engineering, Faculty of Mechanical Engineering, Delft University of Technology, Mekelweg 2, 2628CD Delft, The Netherlands*

⊥*Climate Safety and Security Centre, TU Delft The Hague Campus, Delft University of Technology, 2594 AC, The Hague, The Netherlands*

#*Shell Global Solutions International B.V., Grasweg 39, 1031HW Amsterdam, The Netherlands*

@*Shell Chemical LP, Monaca, PA 15061, The United States*

△*Van 't Hoff Institute of Molecular Sciences, University of Amsterdam, Science Park 904, 1098XH, Amsterdam, The Netherlands*

E-mail: t.j.h.vlugt@tudelft.nl

The following items are presented in this Supporting Information:

1. Force field parameters for alkanes and zeolite used to compute Henry coefficients using molecular simulations

2. Error analysis of Henry coefficients computed from molecular simulations

3. Parity plots for predictions of the negative logarithm of Henry coefficients for linear (C_1-C_{30}) and methyl-branched (C_4-C_{20}) alkanes in MTW-, MTT-, MRE-, and AFI-type zeolites at 523 K using Random Forest (RF), Extreme Gradient Boosting (XGB), Cat Boost (CB), Tabular Prior Fitted Network (TabPFN), and Directed Message Passing Neural Network (D-MPNN).

4. Parity plots for predictions of the negative logarithm of Henry coefficients for linear (C_1-C_{30}) and methyl-, ethyl-, propyl-, and isopropyl-branched (C_4-C_{20}) alkanes in MTW-, MTT-, MRE-, and AFI-type zeolites at 523 K using Random Forest (RF), Extreme Gradient Boosting (XGB), Cat Boost (CB), Tabular Prior Fitted Network (TabPFN), and Directed Message Passing Neural Network (D-MPNN).

5. Reaction equilibrium distribution for hydroisomerization of C_{16} isomers in MTW-type zeolite at 523 K.

6. Bar plots showing average Henry coefficients and average selectivities for different categories of C_{16} isomers relative to linear C_{16} at reaction equilibrium for hydroisomerization in MTW-, MTT-, MRE-, and AFI-type zeolites at 523 K.

Force field parameters for alkanes and zeolite

Table S1: Lennard-Jones force field parameters for united atoms to account for intramolecular and intermolecular non-bonded interactions in alkanes. These parameters are taken from Dubbeldam *et al.*[1]

united atom	ϵ/k_B/ [K]	σ/ [Å]
CH_3	108.0	3.76
CH_2	56.0	3.96
CH	17.0	4.67
C	0.8	6.38

Table S2: Lennard-Jones force field parameters for zeolite atoms to account for intermolecular non-bonded interactions between zeolite atoms and alkanes. These parameters are taken from Bai *et al.*[2]

atom	ϵ/k_B/ [K]	σ/ [Å]
O	53.0	3.30
Si	22.0	2.30

Table S1 lists the Lennard-Jones parameters for the united atoms present in alkanes. These parameters are used to compute intramolecular and intermolecular interactions of alkanes and are taken from Dubbeldam *et al.*[1] To account for the interactions between the zeolite atoms and alkanes, the TraPPE-zeo force field[2] is used. The Lennard-Jones parameters for O and Si are listed in Table S2. For interactions between different types of atoms, the Lennard-Jones parameters are calculated using the Lorentz-Berthelot mixing rules.[3] The TraPPE united atom force field is used for the intramolecular bonded interactions present in alkanes. These interactions are divided into bond-stretching, bond-bending, and torsion potentials. TraPPE force field uses a fixed $\mathrm{C}-\mathrm{C}$ bond length which is equal to 1.54 Å.[2] The bond-bending potential is described by a harmonic potential:[4]

$$U_\mathrm{bend} = \frac{1}{2} k_\mathrm{bend} \left(\theta - \theta_0\right)^2 \tag{S1}$$

In Eq. S1, k_bend is the force constant for the bending potential and θ is the bending angle.

Table S3: Values of the reference angles θ_0 and the force constants k_{bend} for different bend types.

bend type	θ_0/ [deg]	k_{bend}/k_B/ [K/rad^2]
$CH_x - CH_2 - CH_y$	114.0	62500
$CH_x - CH - CH_y$	112.0	62500
$CH_x - C - CH_y$	109.47	62500

Table S4: Values of the coefficients in the TraPPE united atom torsion potential[5,6] for different torsion types.

torsion type	c_0/k_B/ [K]	c_1/k_B/ [K]	c_2/k_B/ [K]	c_3/k_B/ [K]
CH_x-CH_2-CH_2-CH_y	0	355.03	-68.19	791.32
CH_x-CH_2-CH-CH_y	-251.06	428.73	-111.85	441.27
CH_x-CH_2-C-CH_y	0	0	0	461.29
CH_x-CH-CH-CH_y	-251.06	428.73	-111.85	441.27
CH_x-CH-C-CH_y	0	0	0	1635.7
CH_x-C-C-CH_y	0	0	0	1635.7

The values for the reference angles θ_0 are listed in Table S3.[5] These values depend on the type of united atom present at the center of the bend. The torsion potential is described by the three-cosine dihedral equation.[4]

$$U_{\text{torsion}} = c_0 + c_1 [1 + \cos(\phi)] + c_2 [1 - \cos(2\phi)] + c_3 [1 + \cos(3\phi)] \tag{S2}$$

The coefficients (c_0, c_1, c_2, c_3) in Eq. S2 for different types of torsions are listed in Table S4.[5] Table S4 lists the values of the coefficients for different torsions described by the three-cosine dihedral equation. The TraPPE force field[5] parameters for the torsion type CH_x-C-C-CH_y are not available. In this study, this torsion type is obtained from Ref.[6]

Table S5: Henry coefficients and corresponding errors of some linear and mono methyl branched alkanes in MTT-type zeolite at 523 K.

Isomer	Henry coefficient/ [mol/kg/framework]	Error/ [mol/kg/framework]
C_{14}	1.26×10^{-2}	4.78×10^{-4}
3-m-C_8	1.53×10^{-5}	1.11×10^{-7}
6-m-C_{11}	6.97×10^{-4}	3.88×10^{-5}

Table S6: Henry coefficients and corresponding errors of some highly branched alkanes in MTT-type zeolite at 523 K.

Isomer	Henry coefficient/ [mol/kg/framework]	Error/ [mol/kg/framework]
4,4-e-2,2,3,3-m-6-ip-C_9	1.64×10^{-86}	5.04×10^{-55}
4,6-e-2-m-4,5-ip-C_9	2.68×10^{-128}	4.56×10^{-86}
3,3-e-2,4-m-5,5-p-C_8	1.76×10^{-130}	2.78×10^{-62}

Error analysis of Henry coefficients computed from molecular simulations

While error bars are generally useful, including these on the parity plots for CBMC-derived Henry coefficients is not meaningful in this context. For linear and moderately branched alkanes, the CBMC simulations yield small error bars (Table S5) due to efficient sampling and high insertion probabilities. The errors in RASPA are computed as the 95% confidence interval. For long-chain and highly branched alkanes in narrow-pore zeolites, the error bars may become disproportionately large (Table S6). This is a consequence of the poor fit of these molecules inside the zeolite channels. The low values in Table S6 indicate that, at the experimental pressures (ca. 20 bar), these molecules will not adsorb appreciably. Therefore, whether the Henry coefficient is on the order of 10^{-40} or 10^{-80} is irrelevant at 20 bar, they will not fit in the channels regardless. These large uncertainties do not reflect inherent physical variability, but rather the limitations of the Configurational-Bias Monte Carlo method in these extreme cases. Moreover, such bulky isomers are typically of limited relevance for adsorption in narrow-pore zeolites because these isomers simply do not fit well in the zeolite

pores, and including the respective error bars would distract from the overall trends in the data.

Comparison between Henry coefficients for alkanes predicted using different machine learning models

Parity plots for the negative logarithm of Henry coefficients -ln(k_H) for linear (C_1-C_{30}) and methyl-branched (C_4-C_{20}) alkanes predicted by RF,[7] XGB,[8] CB,[9,10] TabPFN,[11,12] and D-MPNN[13–16] are shown in Figs. S1-S4 for MTW-, MTT-, MRE-, and AFI-type zeolites at 523 K. Similarly, parity plots for -ln(k_H) for linear (C_1-C_{30}) and methyl-, ethyl-, propyl-, and isopropyl-branched (C_4-C_{20}) alkanes are shown in Figs. S5-S8 for MTW-, MTT-, MRE-, and AFI-type zeolites at 523 K. In most cases, TabPFN and D-MPNN provide better predictions (larger R^2) compared to the other ML models.

In Fig. S9, the effect of training set size on the accuracy of the D-MPNN model in predicting -ln(k_H) for linear (C_1–C_{30}) and methyl-branched alkanes (C_4–C_{20}) alkanes in MTW- and MTT-type zeolites at 523 K was tested for the active learning and random selection strategies. The initial 50 molecular structures selected by active learning from the training sets, comprising linear alkanes (C_1–C_{30}) and methyl-branched alkanes (C_4–C_{20}), as well as linear alkanes (C_1–C_{30}) and methyl-, ethyl-, propyl-, and isopropyl-branched alkanes (C_4–C_{20}) are shown in Figs. S10.

Activity cliffs present a significant challenge in predicting Henry coefficients of alkanes in zeolites, as minor changes in molecular structure can cause large, order of magnitude variations in adsorption behavior. We examined the effect of oversampling high activity cliff isomers in the training set, as described in Section 2 of the main text. Oversampling 20% of these isomers yielded a modest 4% increase in R^2. Table S7 summarizes the improvements in Henry coefficient predictions for these isomers obtained using the TabPFN model. Expanding the dataset with additional isomers will enhance predictive performance, and complementary

Table S7: Henry coefficients (k_H) of alkanes in zeolites: actual values and predictions from TabPFN model without and with oversampling of high activity cliff isomers.

Isomer	k_H simulation/ [mol/kg/Pa]	k_H predicted/ [mol/kg/Pa] without oversampling	with oversampling
2,7-m-C_{16}	6.185×10^{-3}	3.314×10^{-3}	3.496×10^{-3}
2-m-C_8	2.327×10^{-5}	2.759×10^{-5}	2.621×10^{-5}
3,11-m-C_{18}	5.404×10^{-2}	4.003×10^{-2}	5.308×10^{-2}
4,4-m-C_{16}	5.265×10^{-3}	4.669×10^{-3}	5.133×10^{-3}
2,4-m-C_{18}	3.072×10^{-2}	1.782×10^{-2}	1.793×10^{-2}
3,9-m-C_{11}	8.246×10^{-6}	1.579×10^{-5}	1.389×10^{-5}
3-m-C_{14}	1.629×10^{-3}	2.828×10^{-3}	2.751×10^{-3}
7,8-m-C_{16}	3.357×10^{-4}	1.201×10^{-3}	8.177×10^{-4}
2,2-m-C_9	1.877×10^{-5}	2.153×10^{-5}	2.006×10^{-5}
3,7-m-C_{16}	6.064×10^{-2}	1.379×10^{-2}	1.496×10^{-2}
3,6-m-C_{10}	3.744×10^{-6}	6.667×10^{-6}	6.645×10^{-6}
2,3,5,11,13-m-C_{15}	1.015×10^{-7}	1.944×10^{-8}	2.477×10^{-8}
4,4-m-C_{17}	2.475×10^{-2}	1.147×10^{-2}	1.182×10^{-2}
2,6-m-C_{17}	1.754×10^{-1}	8.092×10^{-2}	9.009×10^{-2}
3,14-m-C_{16}	1.507×10^{-3}	2.321×10^{-3}	2.014×10^{-3}
4,7-m-C_{10}	1.120×10^{-6}	2.227×10^{-6}	2.000×10^{-6}
2-m-C_{16}	4.953×10^{-2}	5.447×10^{-2}	5.275×10^{-2}
8,10-m-C_{18}	8.732×10^{-3}	1.748×10^{-2}	1.680×10^{-2}
2,12-m-C_{13}	2.906×10^{-4}	1.803×10^{-4}	2.004×10^{-4}
C_{11}	8.278×10^{-4}	9.070×10^{-4}	8.803×10^{-4}
2,8-m-C_{16}	1.784×10^{-3}	2.297×10^{-3}	2.109×10^{-3}
2,4-m-C_9	5.612×10^{-6}	5.057×10^{-6}	5.258×10^{-6}
2,2,4,5-m-C_6	1.343×10^{-8}	1.268×10^{-10}	1.498×10^{-10}
2,10-m-C_{18}	4.625×10^{-1}	1.349×10^{-1}	1.404×10^{-1}
3,4-m-C_9	6.550×10^{-7}	5.632×10^{-7}	5.800×10^{-7}
4-m-C_9	3.492×10^{-5}	1.558×10^{-5}	1.607×10^{-5}
5,8-m-C_{15}	1.081×10^{-4}	2.512×10^{-4}	1.984×10^{-4}
4,4,6,6-m-C_9	2.399×10^{-15}	1.586×10^{-9}	3.597×10^{-10}
6-m-C_{18}	2.032×10^{-1}	2.740×10^{-1}	2.384×10^{-1}
3,9-m-C_{12}	1.814×10^{-5}	3.101×10^{-5}	2.698×10^{-5}
8,9-m-C_{18}	1.703×10^{-3}	6.603×10^{-3}	5.272×10^{-3}
2,2,5,5,6,9-m-C_{14}	3.365×10^{-10}	9.575×10^{-14}	1.480×10^{-13}
2,2,4,4,7-m-C_8	8.419×10^{-17}	2.512×10^{-14}	5.188×10^{-15}
3,5-m-C_8	1.564×10^{-6}	1.294×10^{-6}	1.365×10^{-6}
2,8-m-C_{14}	2.159×10^{-4}	3.627×10^{-4}	3.166×10^{-4}
4-m-C_{13}	1.563×10^{-3}	7.999×10^{-4}	8.473×10^{-4}
2,13-m-C_{17}	5.371×10^{-3}	2.026×10^{-2}	1.792×10^{-2}
3,5,6,6,10-m-C_{12}	1.174×10^{-8}	4.924×10^{-10}	7.424×10^{-10}
2,2,4,11,11-m-C_{12}	1.618×10^{-8}	4.459×10^{-9}	7.135×10^{-9}
2,9-m-C_{11}	4.610×10^{-5}	6.231×10^{-5}	5.211×10^{-5}
2,8-m-C_{17}	4.534×10^{-3}	5.363×10^{-3}	4.898×10^{-3}
3,13-m-C_{17}	2.695×10^{-3}	4.725×10^{-3}	4.276×10^{-3}
3,8-m-C_{15}	2.505×10^{-4}	4.814×10^{-4}	4.309×10^{-4}
4,5-m-C_9	8.971×10^{-7}	8.748×10^{-7}	9.059×10^{-7}
3,10-m-C_{14}	1.267×10^{-4}	2.531×10^{-4}	2.105×10^{-4}
3,7-m-C_{15}	1.116×10^{-2}	5.460×10^{-3}	5.858×10^{-3}
4,5-m-C_{12}	6.873×10^{-6}	1.265×10^{-5}	1.235×10^{-5}
3,5-m-C_{10}	1.213×10^{-5}	8.660×10^{-6}	9.023×10^{-6}
4,14-m-C_{17}	1.726×10^{-3}	4.744×10^{-3}	3.597×10^{-3}
5,10-m-C_{15}	1.542×10^{-3}	9.916×10^{-4}	1.010×10^{-3}
3,4-m-C_{11}	2.496×10^{-6}	4.008×10^{-6}	3.792×10^{-6}
6-m-C_{17}	7.619×10^{-2}	1.061×10^{-1}	1.008×10^{-1}
7,8-m-C_{15}	8.732×10^{-5}	3.336×10^{-4}	2.531×10^{-4}
2,2,4,7-m-C_9	7.476×10^{-9}	2.158×10^{-9}	2.529×10^{-9}
3,3-m-C_{12}	3.800×10^{-4}	1.100×10^{-4}	1.139×10^{-4}
2,14-m-C_{18}	3.061×10^{-1}	8.094×10^{-2}	8.388×10^{-2}

approaches such as contrastive learning should be explored.

Reaction equilibrium distribution for hydroisomerization of C_{16} isomers

Selectivities of C_{16} isomers relative to n-C16 at reaction equilibrium in MTW-type zeolite are shown in Fig. S11. Mono-methyl isomers show larger selectivities than di-methyl isomers. The high selectivity of 2-m-C_{15} relative to n-C_{16} is attributed to its larger gas-phase mole fraction. For di-methyl alkanes, isomers with methyl groups positioned farther apart (e.g., 2,6-m-C_{14} to 2,13-m-C_{14}) are favored compared to those with closely spaced methyl groups (e.g., 2,2-m-C_{14} to 2,5-m-C_{14}), likely due to reduced steric hindrance in the zeolite pores. The raw data for the reaction equilibrium distribution of C_{16} isomers are provided in SI2_HC.xlsx. Average Henry coefficients for different groups of C_{16} isomers and average selectivities of these groups relative to linear C_{16} in MTW-, MTT-, MRE-, and AFI-type zeolites are shown in Figs. S12-S15. Linear, mono-, di-, tri-, and tetra-methyl, and mono-ethyl isomers are the most favorable groups in terms of relative selectivities in all zeolites considered in this study. Highly branched isomers such as those with both ethyl and propyl groups adsorb poorly in these zeolites (Figs. S12-S15), reflected by low Henry coefficients.

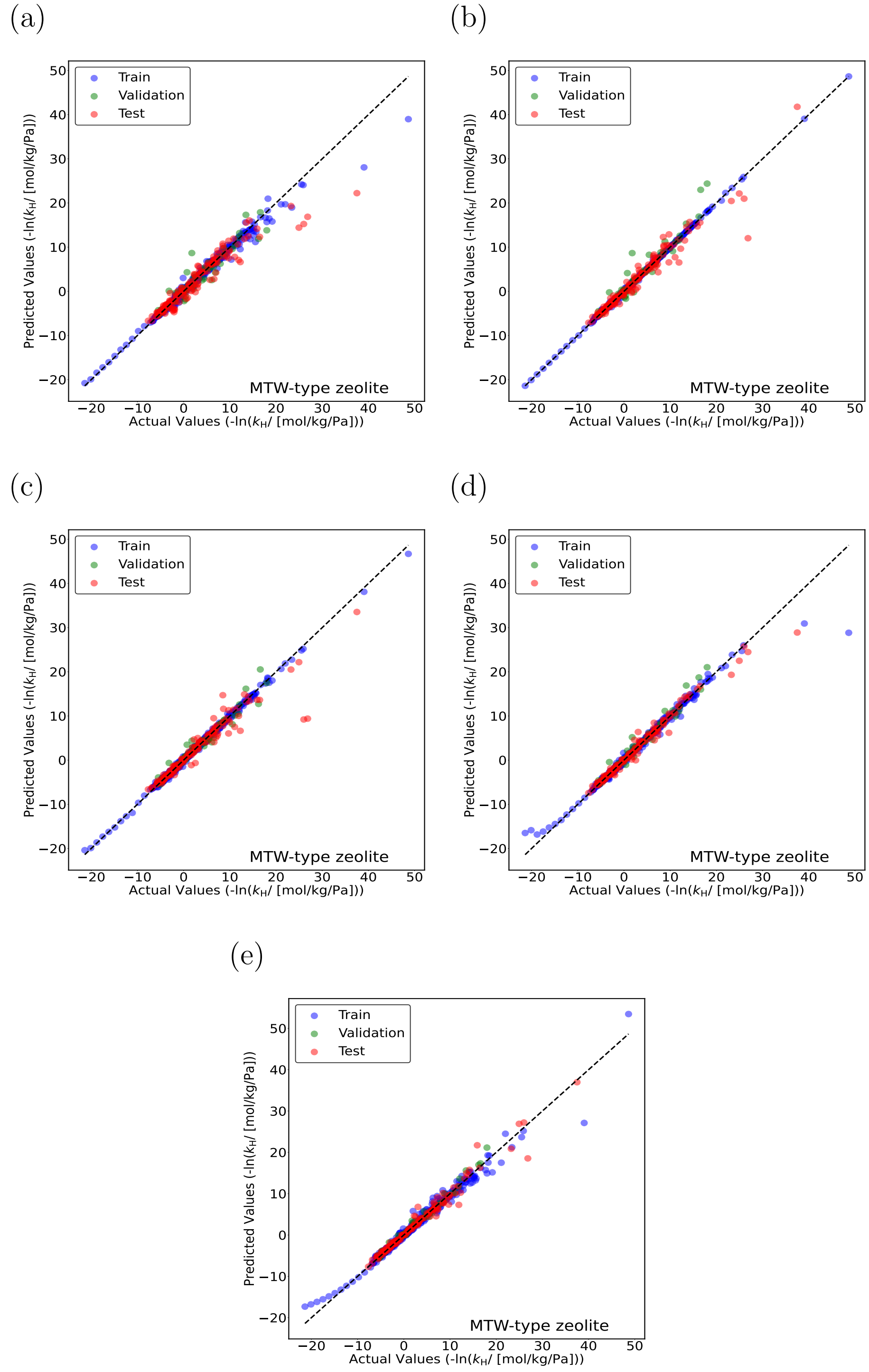

Figure S1: Parity plots for predicting the negative logarithm of Henry coefficients -ln(k_{H}) for linear alkanes (C_1–C_{30}) and methyl-branched alkanes (C_4–C_{20}) in MTW-type zeolite at 523 K using (a) RF, (b) XGB, (c) CB, (d) TabPFN, and (e) D-MPNN models. Blue circles indicate training isomers and green circles represent validation isomers. The random seed is varied to identify the most suitable split between the training and the validation datasets. Red circles represent the test isomers which are never part of the training set. The standard deviations for the actual values of -ln(k_{H}) are too small to plot.

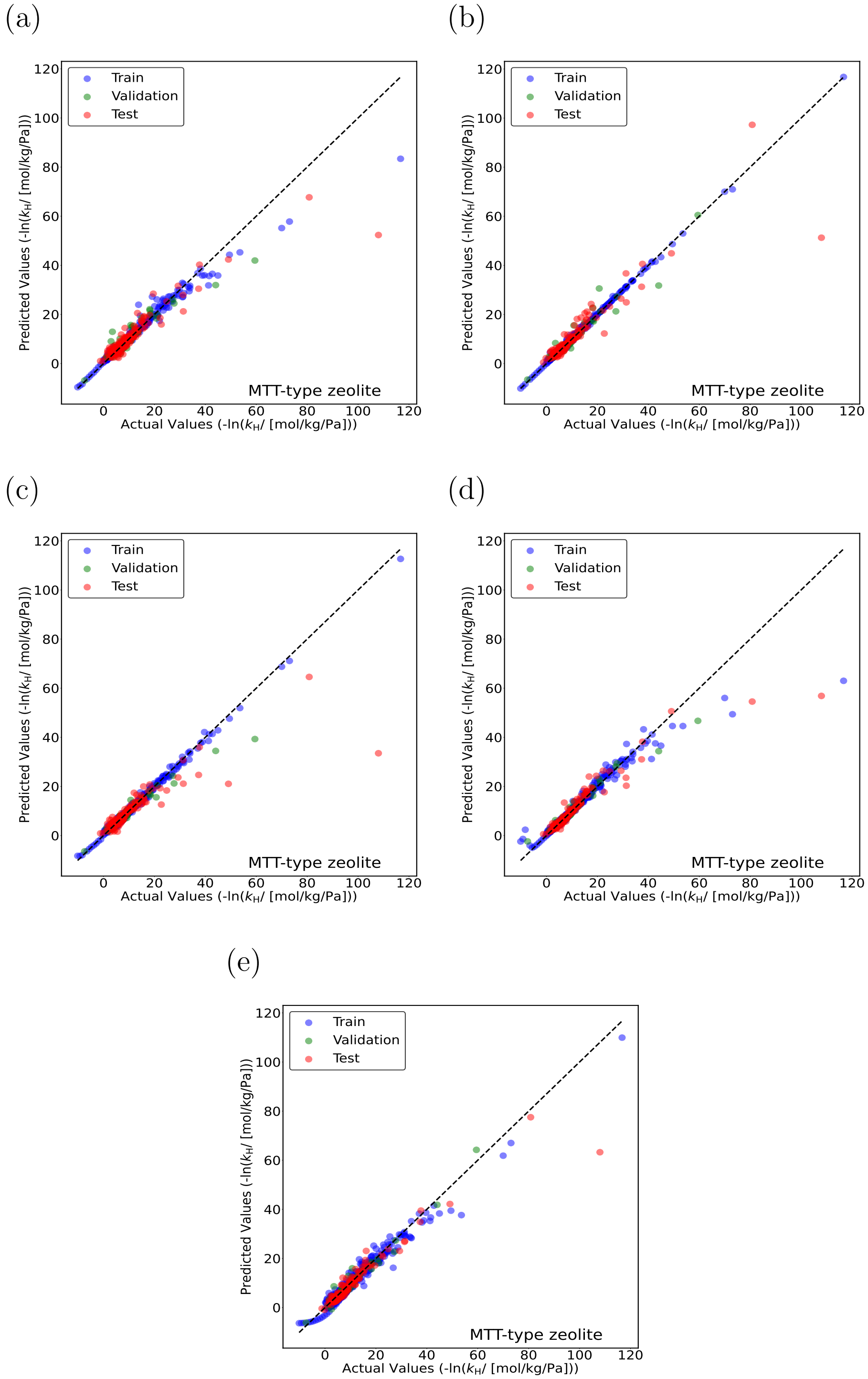

Figure S2: Parity plots for predicting the negative logarithm of Henry coefficients -ln(k_H) for linear alkanes (C_1–C_{30}) and methyl-branched alkanes (C_4–C_{20}) in MTT-type zeolite at 523 K using (a) RF, (b) XGB, (c) CB, (d) TabPFN, and (e) D-MPNN models. Blue circles indicate training isomers and green circles represent validation isomers. The random seed is varied to identify the most suitable split between the training and the validation datasets. Red circles represent the test isomers which are never part of the training set. The standard deviations for the actual values of -ln(k_H) are too small to plot.

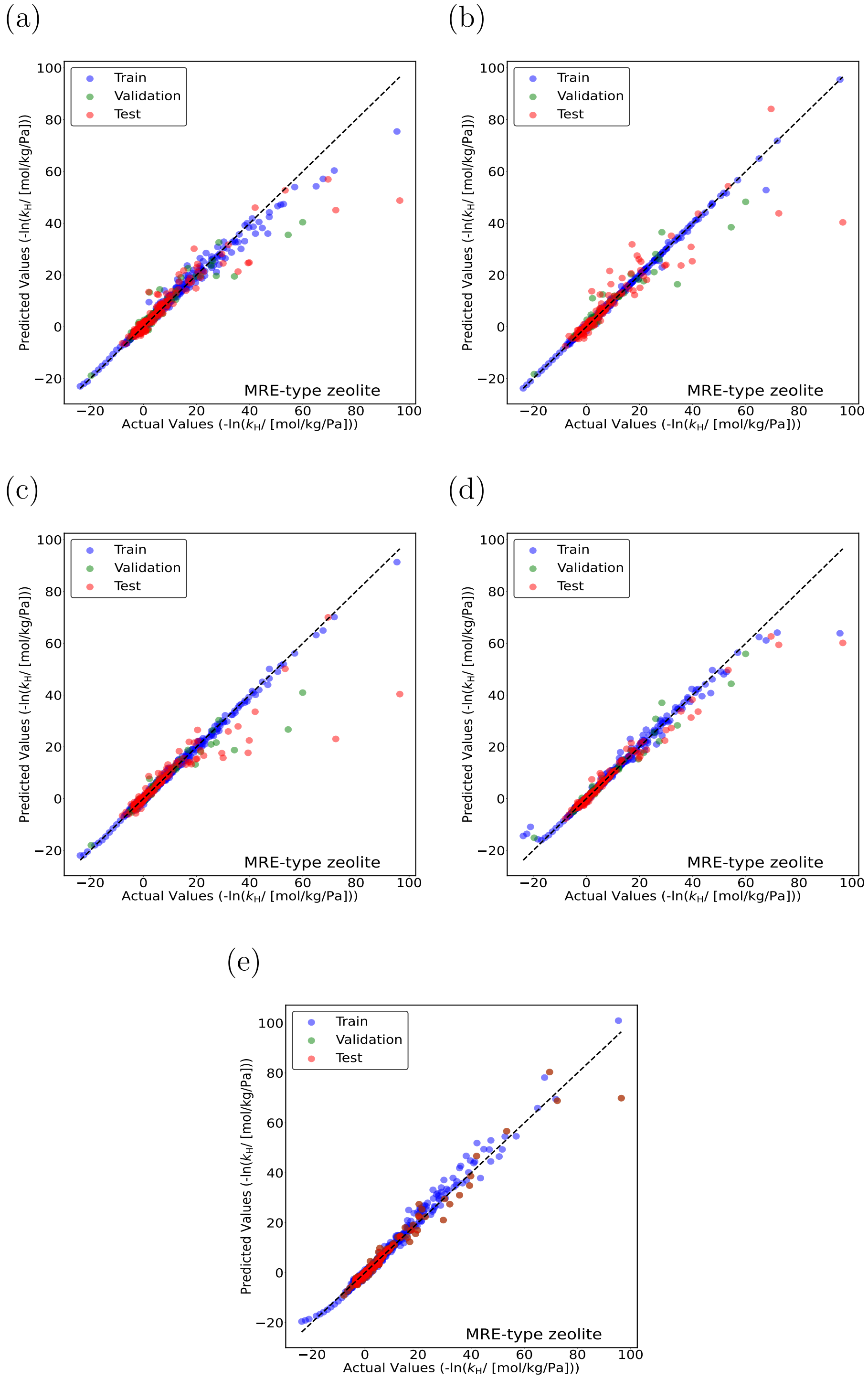

Figure S3: Parity plots for predicting the negative logarithm of Henry coefficients -ln(k_H) for linear alkanes (C_1–C_{30}) and methyl-branched alkanes (C_4–C_{20}) in MRE-type zeolite at 523 K using (a) RF, (b) XGB, (c) CB, (d) TabPFN, and (e) D-MPNN models. Blue circles indicate training isomers and green circles represent validation isomers. The random seed is varied to identify the most suitable split between the training and the validation datasets. Red circles represent the test isomers which are never part of the training set. The standard deviations for the actual values of -ln(k_H) are too small to plot.

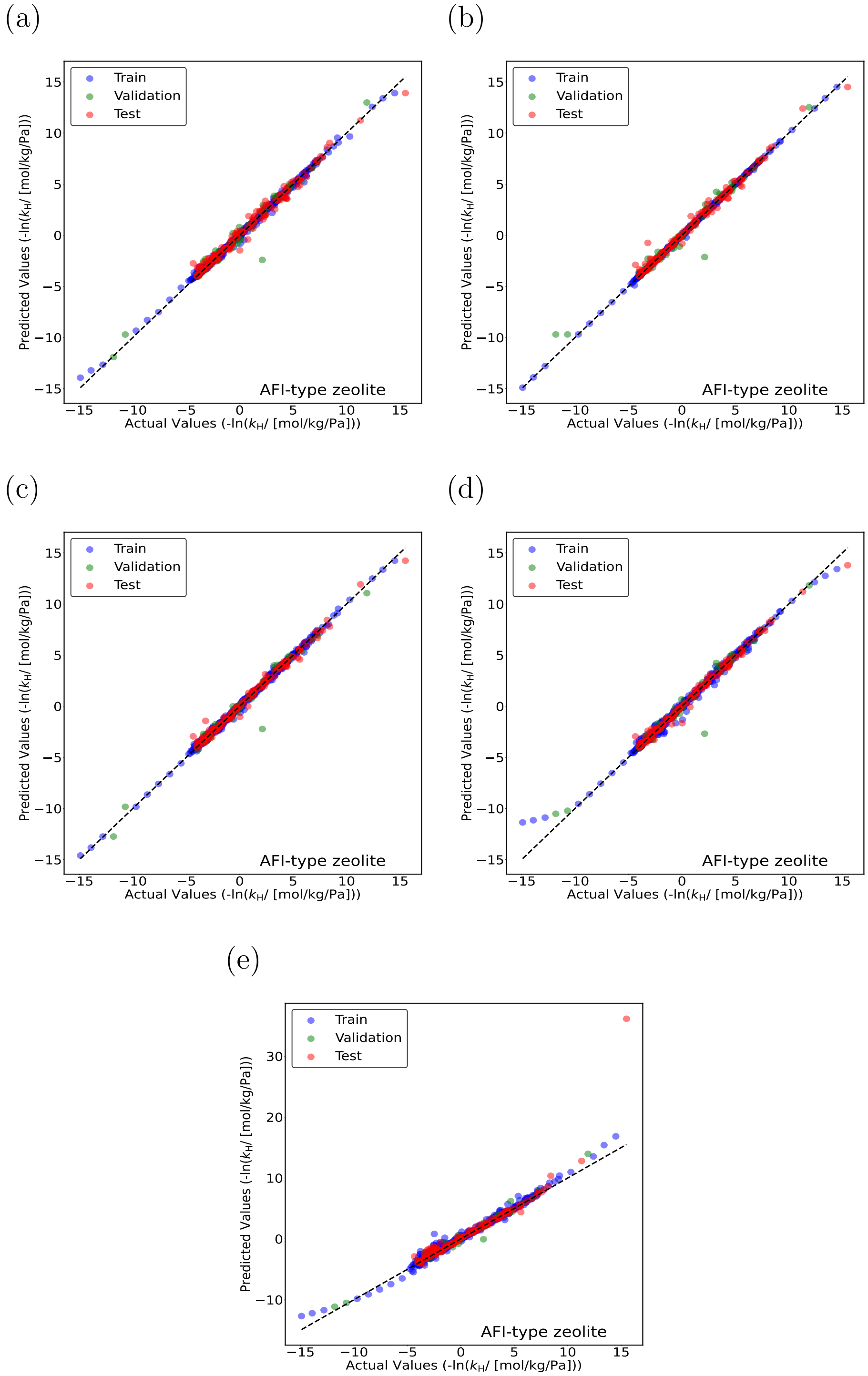

Figure S4: Parity plots for predicting the negative logarithm of Henry coefficients -ln(k_H) for linear alkanes (C_1–C_{30}) and methyl-branched alkanes (C_4–C_{20}) in AFI-type zeolite at 523 K using (a) RF, (b) XGB, (c) CB, (d) TabPFN, and (e) D-MPNN models. Blue circles indicate training isomers and green circles represent validation isomers. The random seed is varied to identify the most suitable split between the training and the validation datasets. Red circles represent the test isomers which are never part of the training set. The standard deviations for the actual values of -ln(k_H) are too small to plot.

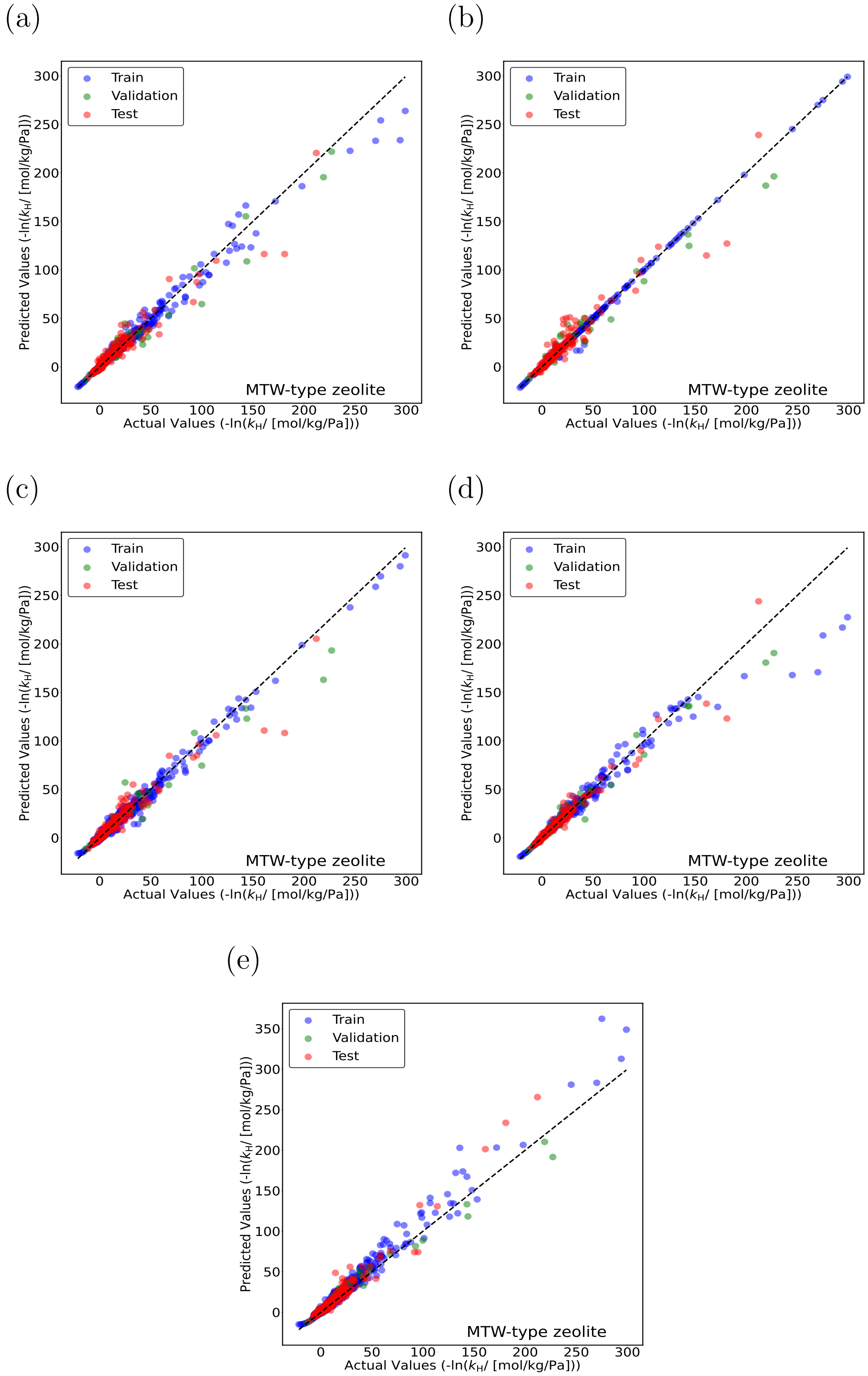

Figure S5: Parity plots for predicting -ln(k_H), for linear (C_1–C_{30}) and methyl-, ethyl-, propyl-, and isopropyl-branched alkanes (C_4–C_{20}) in MTW-type zeolite at 523 K using (a) RF, (b) XGB, (c) CB, (d) TabPFN, and (e) D-MPNN models. Blue circles indicate training isomers and green circles represent validation isomers. The random seed is varied to identify the most suitable split between the training and the validation datasets. Red circles represent the test isomers which are never part of the training set. The standard deviations for the actual values of -ln(k_H) are too small to plot.

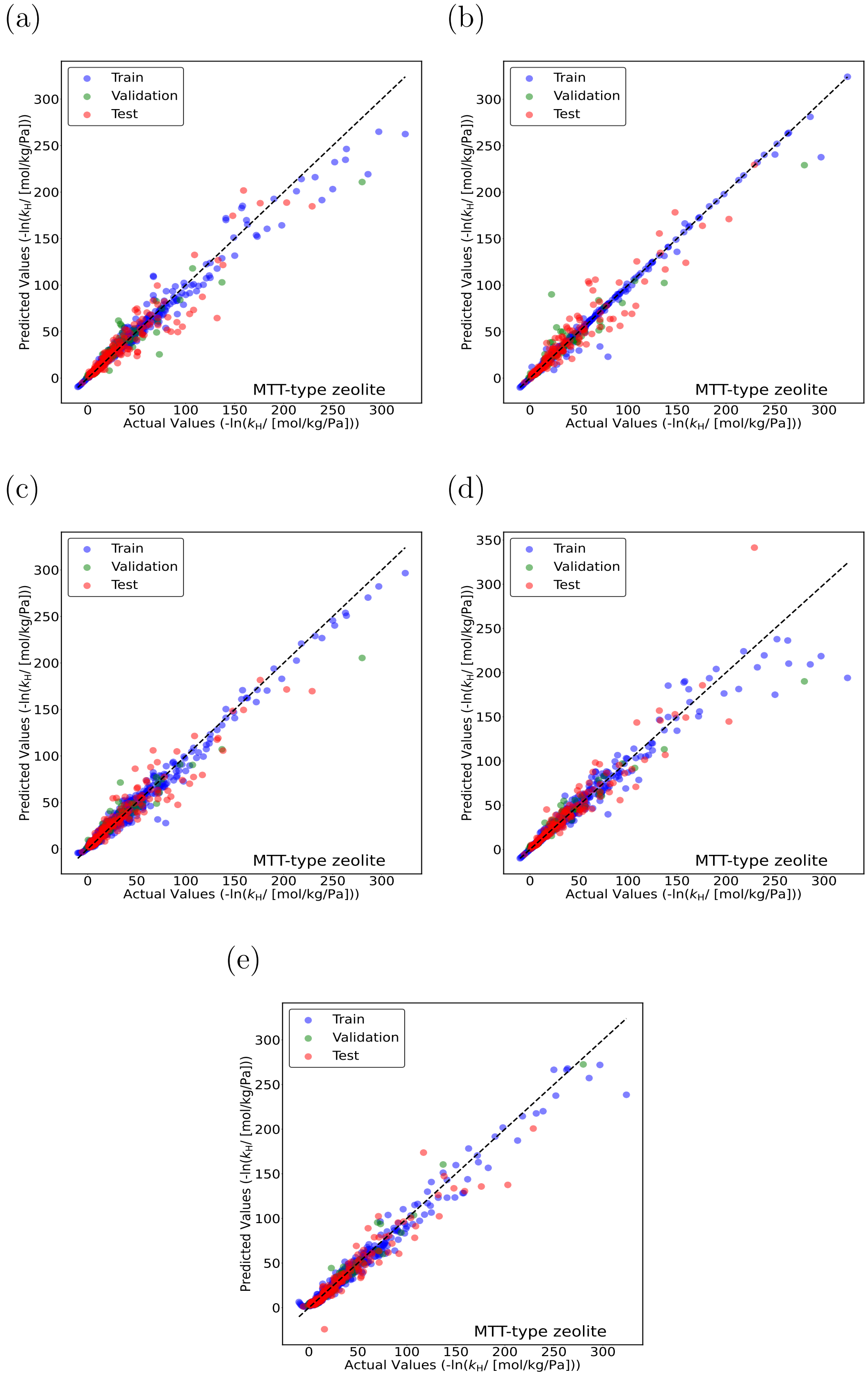

Figure S6: Parity plots for predicting -ln(k_H), for linear (C_1–C_{30}) and methyl-, ethyl-, propyl-, and isopropyl-branched alkanes (C_4–C_{20}) in MTT-type zeolite at 523 K using (a) RF, (b) XGB, (c) CB, (d) TabPFN, and (e) D-MPNN models. Blue circles indicate training isomers, and green circles represent validation isomers. The random seed is varied to identify the most suitable split between the training and the validation datasets. Red circles represent the test isomers, which are never part of the training set. The standard deviations for the actual values of -ln(k_H) are too small to plot.

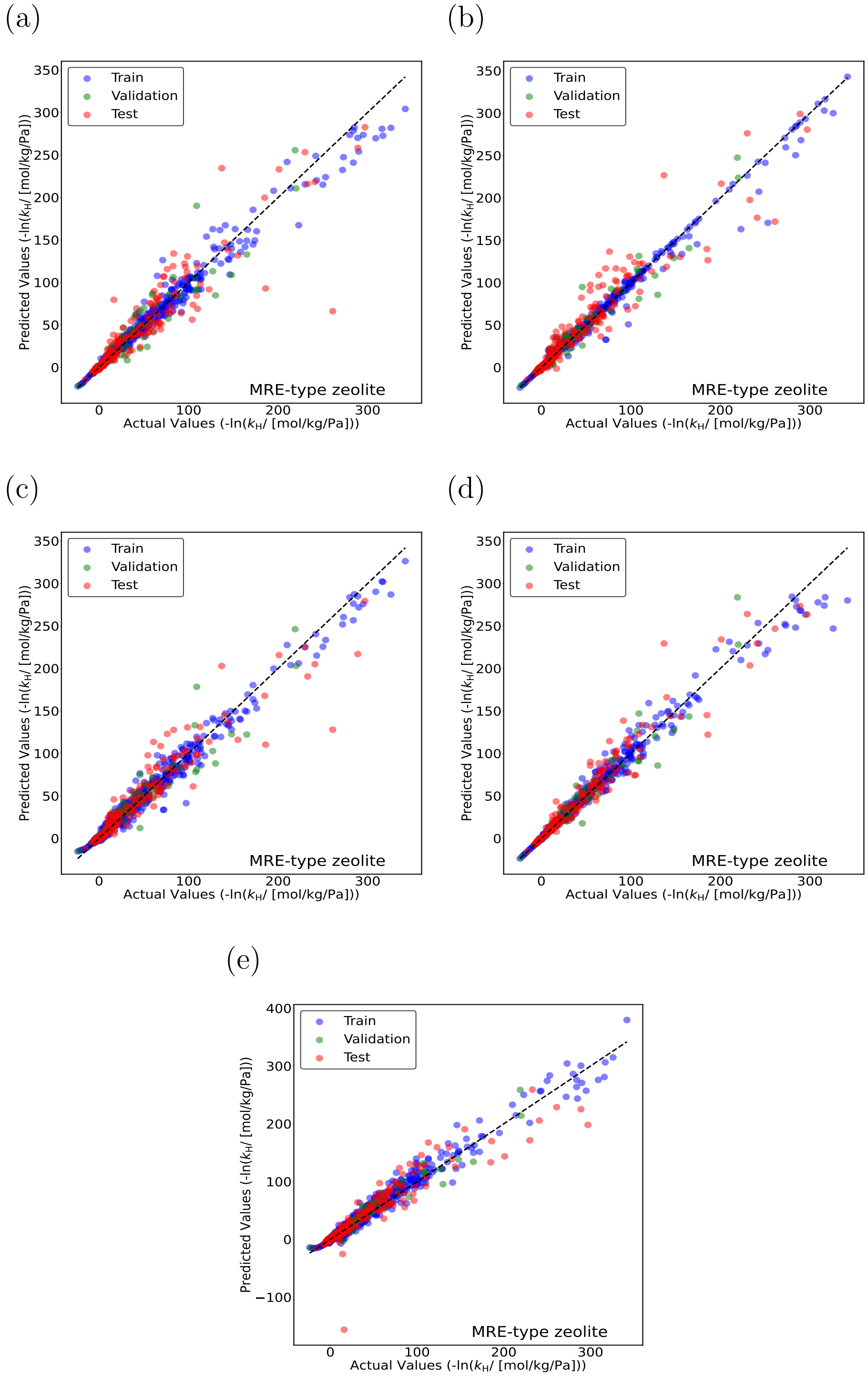

Figure S7: Parity plots for predicting -ln(k_H), for linear (C_1–C_{30}) and methyl-, ethyl-, propyl-, and isopropyl-branched alkanes (C_4–C_{20}) in MRE-type zeolite at 523 K using (a) RF, (b) XGB, (c) CB, (d) TabPFN, and (e) D-MPNN models. Blue circles indicate training isomers and green circles represent validation isomers. The random seed is varied to identify the most suitable split between the training and the validation datasets. Red circles represent the test isomers which are never part of the training set. The standard deviations for the actual values of -ln(k_H) are too small to plot.

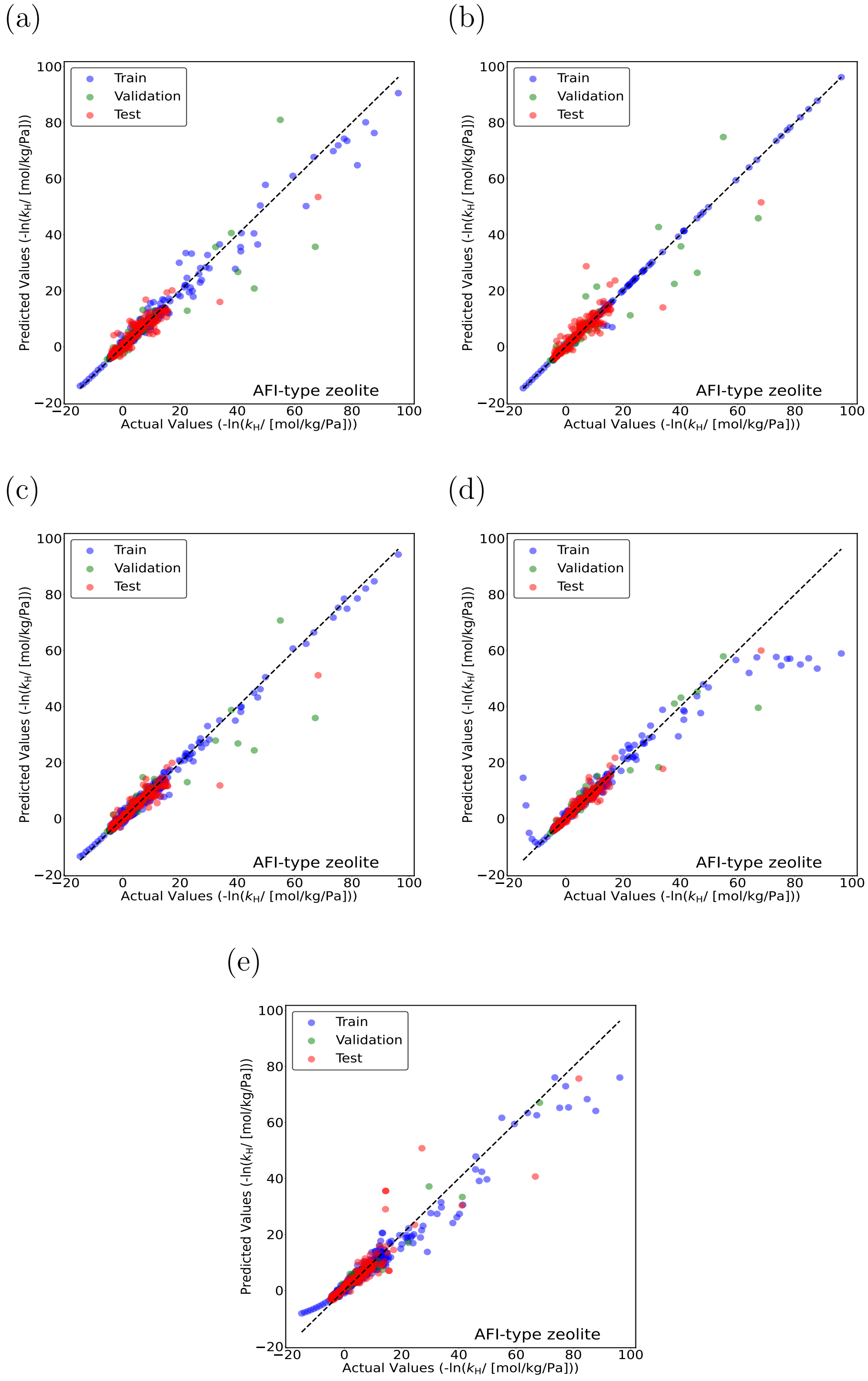

Figure S8: Parity plots for predicting -ln(k_H), for linear (C_1–C_{30}) and methyl-, ethyl-, propyl-, and isopropyl-branched alkanes (C_4–C_{20}) in AFI-type zeolite at 523 K using (a) RF, (b) XGB, (c) CB, (d) TabPFN, and (e) D-MPNN models. Blue circles indicate training isomers and green circles represent validation isomers. The random seed is varied to identify the most suitable split between the training and the validation datasets. Red circles represent the test isomers which are never part of the training set. The standard deviations for the actual values of -ln(k_H) are too small to plot.

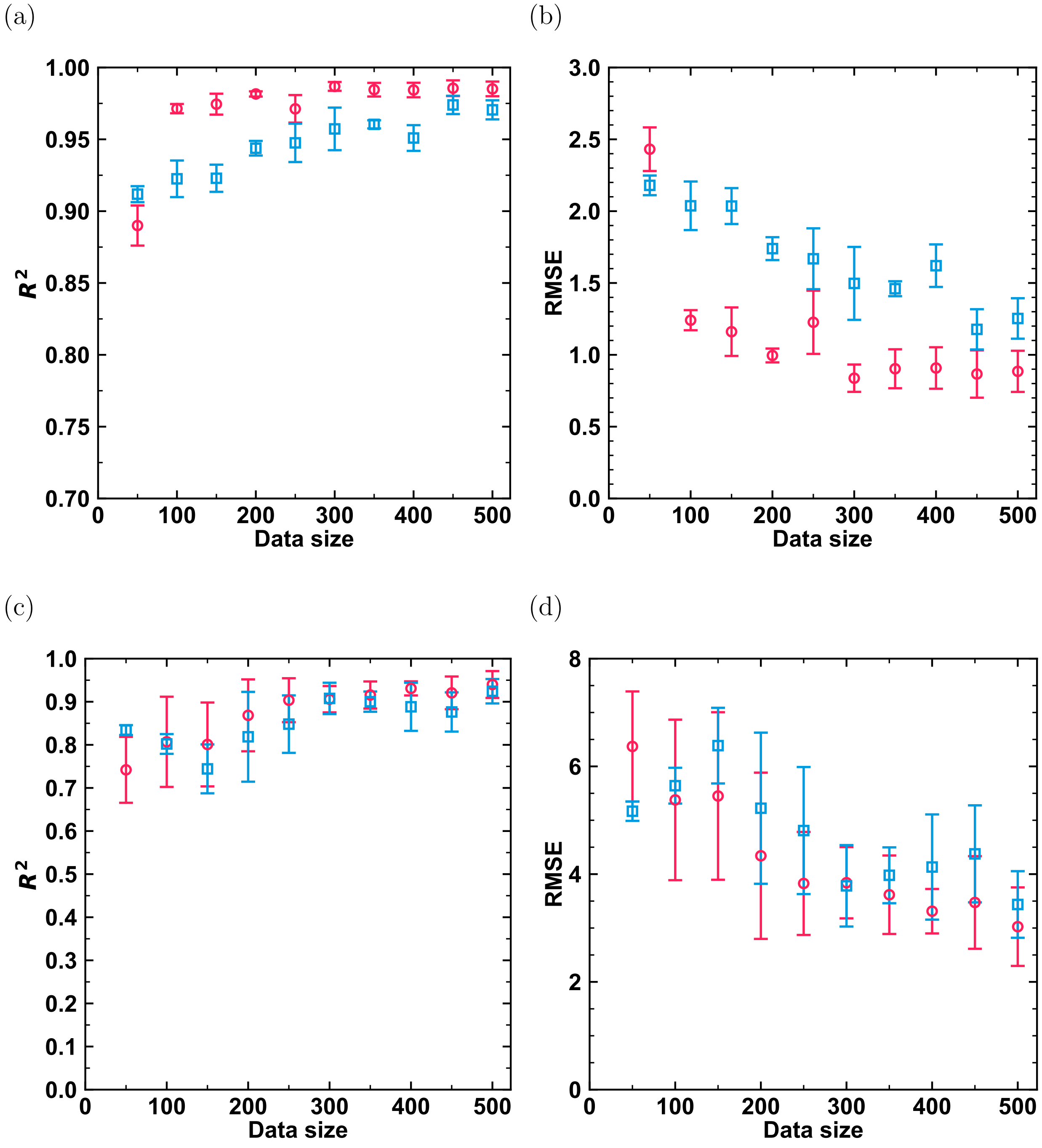

Figure S9: Test accuracies of the D-MPNN model as a function of training set size, comparing an active learning strategy (red circles) and a random selection strategy (blue squares). R^2 and RMSE values are shown for models trained on the negative logarithm of Henry coefficients for linear (C_1–C_{30}) and methyl-branched (C_4–C_{20}) alkanes in MTW-type (a, b) and MTT-type (c, d) zeolites at 523 K.

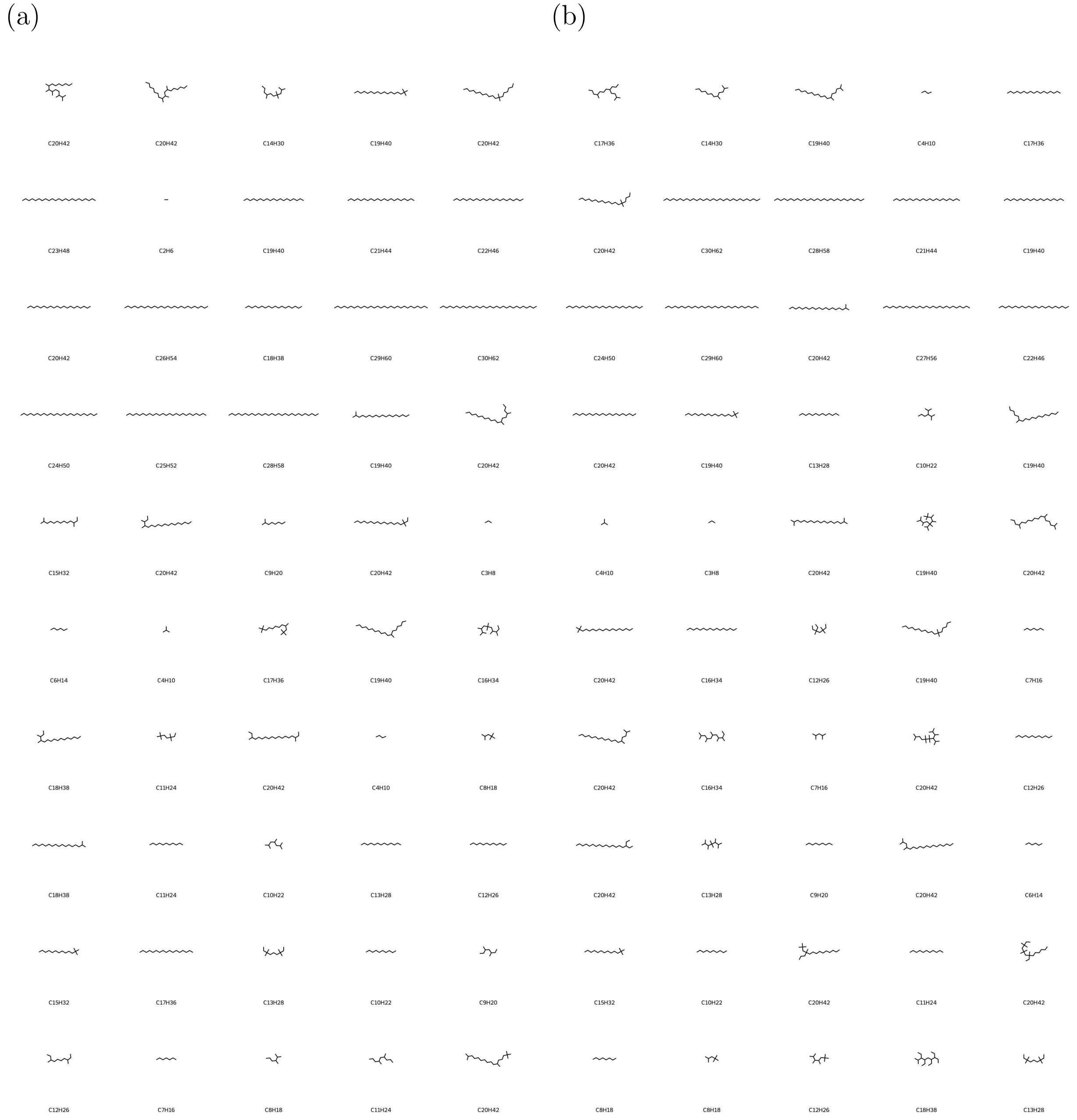

Figure S10: Initial 50 molecular structures selected by active learning from a training set of (a) linear (C_1–C_{30}) and methyl-branched alkanes (C_4–C_{20}) and (b) linear (C_1–C_{30}) and methyl-, ethyl-, propyl-, and isopropyl-branched alkanes (C_4–C_{20}).

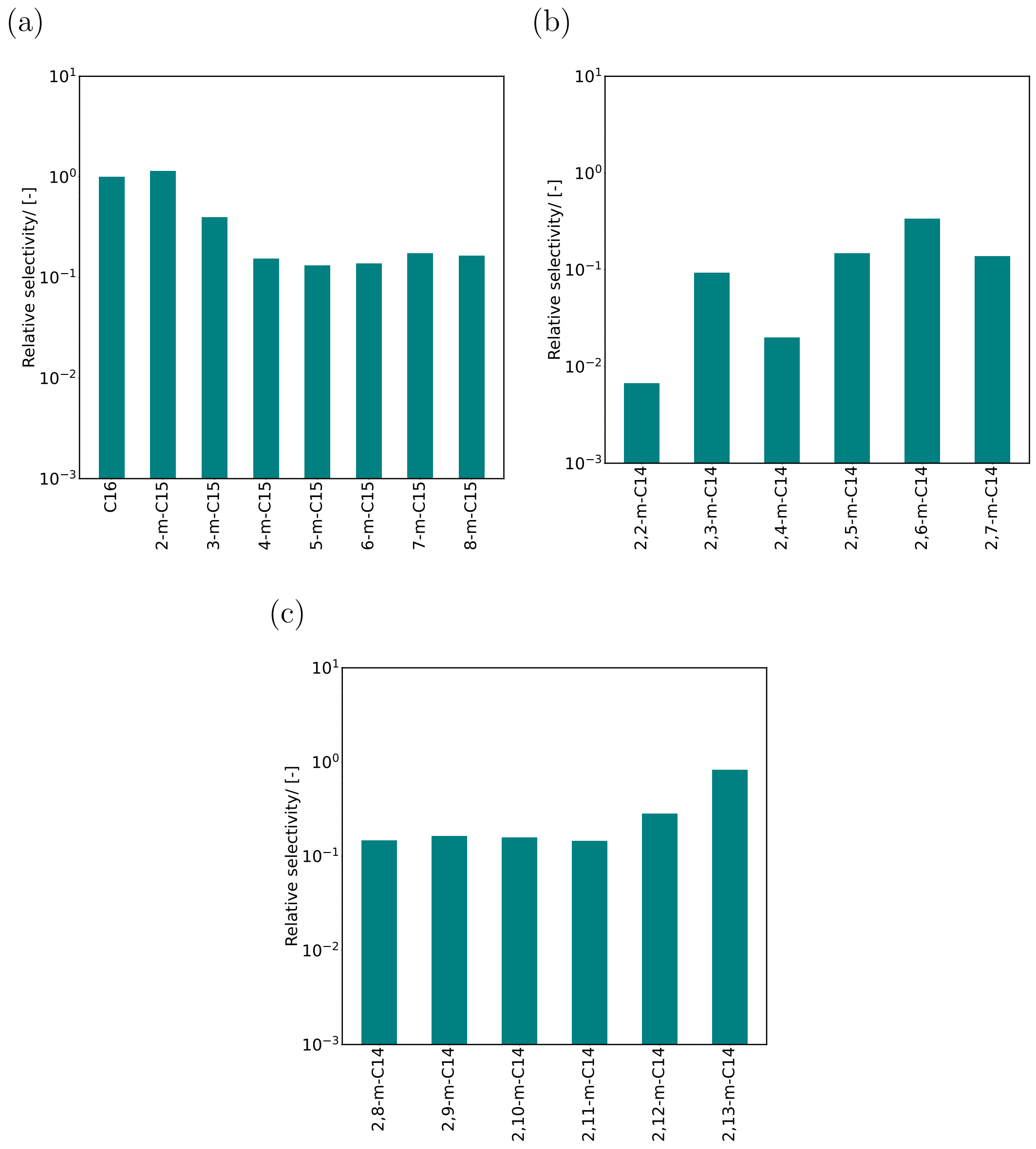

Figure S11: Selectivities of (a) n-C_{16} and mono-methyl (2-m-C_{15} - 8-m-C_{15}), (b) di-methyl (2,2-m-C_{14} - 2,7-m-C_{14}), and (c) di-methyl (2,8-m-C_{14} - 2,13-m-C_{14}) isomers relative to n-C_{16} at reaction equilibrium in MTW-type zeolite at infinite dilution and 523 K. The absolute selectivities are defined as the mole fraction of a certain component divided by the sum of the mole fractions of all other components.[17] The relative selectivities refer to the ratio of the absolute selectivity of a specific isomer to that of the reference isomer, which is C_{16} in this case.

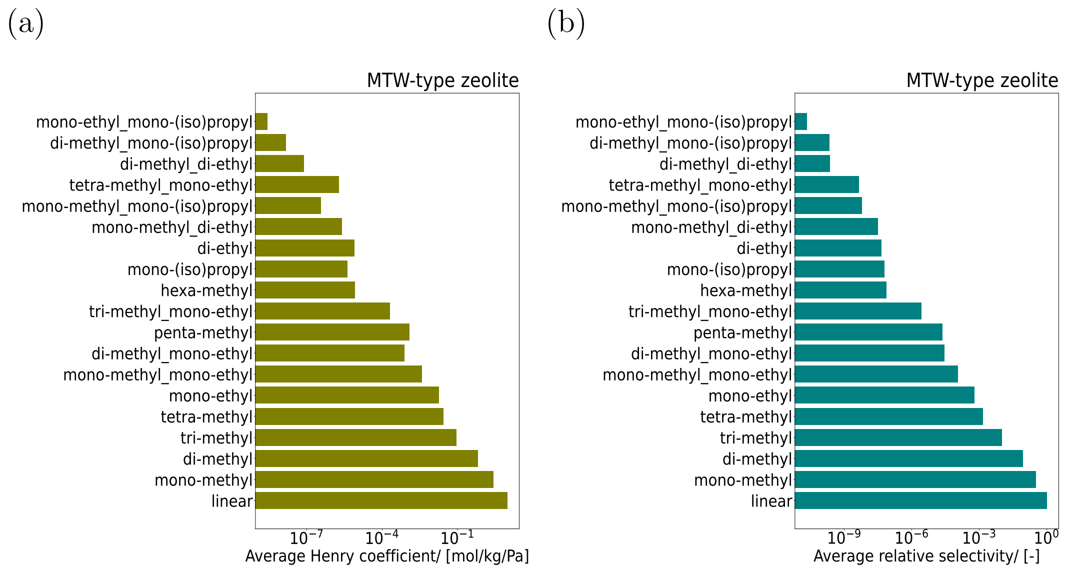

Figure S12: (a) Average Henry coefficients for different categories of C_{16} isomers in MTW-type zeolite at 523 K predicted using the TabPFN model. (b) Average selectivities of different categories of C_{16} isomers relative to linear C_{16} at reaction equilibrium in MTW-type zeolite at 523 K. For each category, the Henry coefficients and the selectivities are averaged over all the isomers belonging to that category.

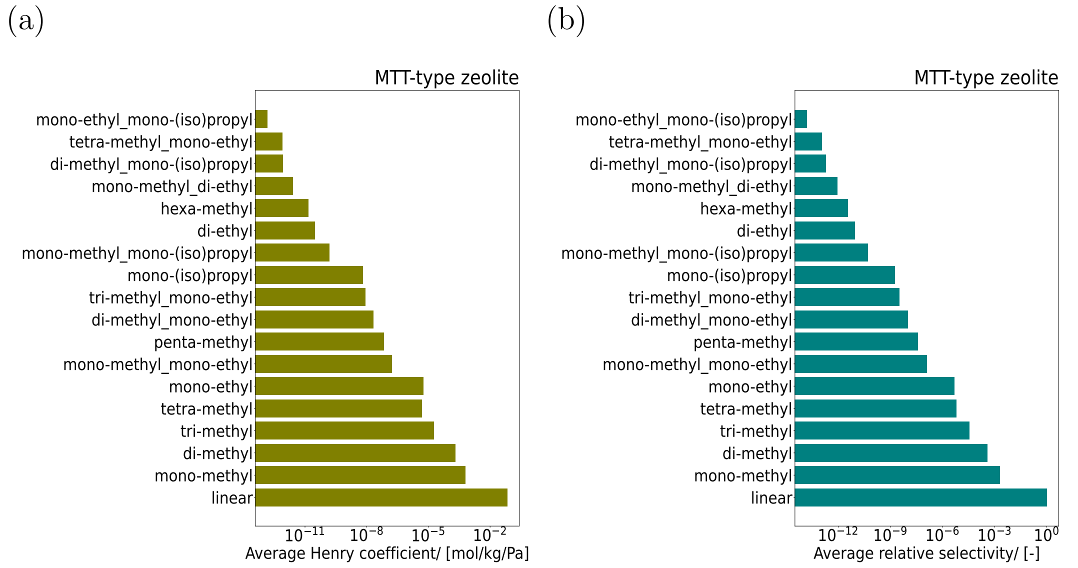

Figure S13: (a) Average Henry coefficients for different categories of C_{16} isomers in MTT-type zeolite at 523 K predicted using the TabPFN model. (b) Average selectivities of different categories of C_{16} isomers relative to linear C_{16} at reaction equilibrium in MTT-type zeolite at 523 K. For each category, the Henry coefficients and the selectivities are averaged over all the isomers belonging to that category.

(a) (b)

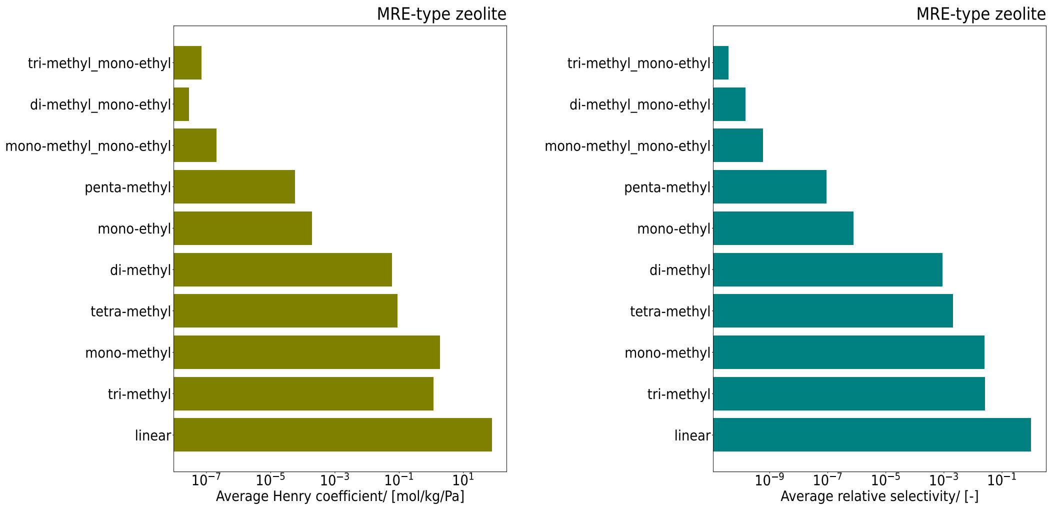

Figure S14: (a) Average Henry coefficients for different categories of C_{16} isomers in MRE-type zeolite at 523 K predicted using the TabPFN model. (b) Average selectivities of different categories of C_{16} isomers relative to linear C_{16} at reaction equilibrium in MRE-type zeolite at 523 K. For each category, the Henry coefficients and the selectivities are averaged over all the isomers belonging to that category.

(a) (b)

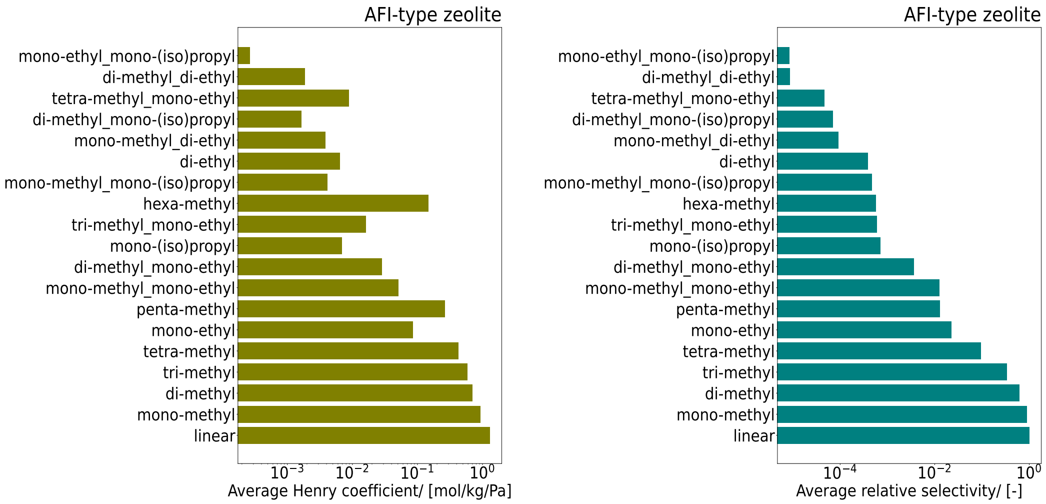

Figure S15: (a) Average Henry coefficients for different categories of C_{16} isomers in AFI-type zeolite at 523 K predicted using the TabPFN model. (b) Average selectivities of different categories of C_{16} isomers relative to linear C_{16} at reaction equilibrium in AFI-type zeolite at 523 K. For each category, the Henry coefficients and the selectivities are averaged over all the isomers belonging to that category.

References

(1) Dubbeldam, D.; Calero, S.; Vlugt, T. J. H.; Krishna, R.; Maesen, T. L. M.; Smit, B. United atom force field for alkanes in nanoporous materials. *J. Phys. Chem. B* **2004**, *108*, 12301–12313.

(2) Bai, P.; Tsapatsis, M.; Siepmann, J. I. TraPPE-zeo: Transferable potentials for phase equilibria force field for all-silica zeolites. *J. Phys. Chem. C* **2013**, *117*, 24375–24387.

(3) Allen, M. P.; Tildesley, D. J. *Computer simulation of liquids*, 2nd ed.; Oxford University Press: Oxford, 2017.

(4) Lewars, E. G. *Computational Chemistry. Introduction to the Theory and Applications of Molecular and Quantum Mechanics*, 2nd ed.; Springer: New York, 2011.

(5) Martin, M. G.; Siepmann, J. I. Transferable potentials for phase equilibria. 1. United-atom description of n-alkanes. *J. Phys. Chem. B* **1998**, *102*, 2569–2577.

(6) Nath, S. K.; Khare, R. New forcefield parameters for branched hydrocarbons. *J. Chem. Phys.* **2001**, *115*, 10837–10844.

(7) Breiman, L. Random Forests. *Machine Learning* **2001**, *45*, 5–32.

(8) Chen, T.; Guestrin, C. XGBoost: A Scalable Tree Boosting System. Proceedings of the 22nd ACM SIGKDD International Conference on Knowledge Discovery and Data Mining. 2016; pp 785–794.

(9) Prokhorenkova, L.; Gusev, G.; Vorobev, A.; Dorogush, A. V.; Gulin, A. CatBoost: Unbiased Boosting with Categorical Features. Advances in Neural Information Processing Systems 31 (NeurIPS 2018). 2018; https://arxiv.org/abs/1706.09516.

(10) Dorogush, A. V.; Ershov, V.; Gulin, A. CatBoost: gradient boosting with categorical features support. *arXiv preprint arXiv:1810.11363* **2018**,

(11) Hollmann, N.; Müller, S.; Eggensperger, K.; Hutter, F. TabPFN: A transformer that solves small tabular classification problems in a second. The Eleventh International Conference on Learning Representations. 2023; https://doi.org/10.48550/arXiv.2207.01848.

(12) Hollmann, N.; Müller, S.; Purucker, L.; Krishnakumar, A.; Körfer, M.; Hoo, S. B.; Schirrmeister, R. T.; Hutter, F. Accurate predictions on small data with a tabular foundation model. *Nature* **2025**, *637*, 319–326.

(13) Yang, K.; Swanson, K.; Jin, W.; Coley, C.; Eiden, P.; Gao, H.; Guzman-Perez, A.; Hopper, T.; Kelley, B.; Mathea, M.; others Analyzing learned molecular representations for property prediction. *J. Chem. Inf. Model.* **2019**, *59*, 3370–3388.

(14) Heid, E.; Green, W. H. Machine learning of reaction properties via learned representations of the condensed graph of reaction. *J. Chem. Inf. Model.* **2021**, *62*, 2101–2110.

(15) Jin, W.; Barzilay, R.; Jaakkola, T. Multi-objective molecule generation using interpretable substructures. 37th International Conference on Machine Learning. 2020; https://arxiv.org/abs/2002.03244.

(16) Heid, E.; Greenman, K. P.; Chung, Y.; Li, S.-C.; Graff, D. E.; Vermeire, F. H.; Wu, H.; Green, W. H.; McGill, C. J. Chemprop: a machine learning package for chemical property prediction. *J. Chem. Inf. Model.* **2023**, *64*, 9–17.

(17) Levenspiel, O. *Chemical reaction engineering*, 3rd ed.; John Wiley & Sons: New York, 1998.
